# Withanolide A Prevents Neurodegeneration by Modulating Hippocampal Glutathione Biosynthesis during Hypoxia

**DOI:** 10.1371/journal.pone.0105311

**Published:** 2014-10-13

**Authors:** Iswar Baitharu, Vishal Jain, Satya Narayan Deep, Sabita Shroff, Jayanta Kumar Sahu, Pradeep Kumar Naik, Govindasamy Ilavazhagan

**Affiliations:** 1 Department of Zoology, Guru Ghasidas Central University, Bilaspur, Chattishgarh, India; 2 Department of Neurobiology, Defence Institute of Physiology and Allied Sciences, Defense Research Development Organisation, Timarpur, Delhi, India; 3 Department of Research, Hindustan University, Chennai, Tamilnadu, India; 4 Department of Life Science, National Institute of Technology, Rourkela, India; 5 Department of Chemistry, Sambalpur University, Burla, India; University of Udine, Italy

## Abstract

*Withania somnifera* root extract has been used traditionally in ayurvedic system of medicine as a memory enhancer. Present study explores the ameliorative effect of withanolide A, a major component of withania root extract and its molecular mechanism against hypoxia induced memory impairment. Withanolide A was administered to male Sprague Dawley rats before a period of 21 days pre-exposure and during 07 days of exposure to a simulated altitude of 25,000 ft. Glutathione level and glutathione dependent free radicals scavenging enzyme system, ATP, NADPH level, γ-glutamylcysteinyl ligase (GCLC) activity and oxidative stress markers were assessed in the hippocampus. Expression of apoptotic marker caspase 3 in hippocampus was investigated by immunohistochemistry. Transcriptional alteration and expression of GCLC and Nuclear factor (erythroid-derived 2)–related factor 2 (Nrf2) were investigated by real time PCR and immunoblotting respectively. Exposure to hypobaric hypoxia decreased reduced glutathione (GSH) level and impaired reduced gluatathione dependent free radical scavenging system in hippocampus resulting in elevated oxidative stress. Supplementation of withanolide A during hypoxic exposure increased GSH level, augmented GSH dependent free radicals scavenging system and decreased the number of caspase and hoescht positive cells in hippocampus. While withanolide A reversed hypoxia mediated neurodegeneration, administration of buthionine sulfoximine along with withanolide A blunted its neuroprotective effects. Exogenous administration of corticosterone suppressed Nrf2 and GCLC expression whereas inhibition of corticosterone synthesis upregulated Nrf2 as well as GCLC. Thus present study infers that withanolide A reduces neurodegeneration by restoring hypoxia induced glutathione depletion in hippocampus. Further, Withanolide A increases glutathione biosynthesis in neuronal cells by upregulating GCLC level through Nrf2 pathway in a corticosterone dependenet manner.

## Introduction

Prolonged exposure to hypobaric hypoxia at high altitude is known to cause hippocampal neurodegeneration leading to loss of memory and higher order brain dysfunctions [1]–[2]. Under hypoxic conditions, lower availability of oxygen at tissue level results in generation of superoxide radicals that subsequently generate hydroxyl and peroxynitrite radicals in a chain reaction [3]. The antioxidants and free radical scavenging enzyme system play a crucial role in quenching the free radicals generated as a byproduct of various biochemical reactions under normoxic condition. However, hypoxic exposure weakens the antioxidant defense mechanisms by causing alterations in activity of antioxidant enzymes like glutathione reductase and glutathione peroxidase [4]–[5]. The cumulative effect of impaired antioxidant system and increased free radical generation leads to lipid peroxidation, membrane damage, protein oxidation, DNA damage [6] and altered gene expression [7] that may finally culminate in cell death. The brain is vulnerable to oxidative stress because of its high demand for oxygen, abundant fatty acids that are targets of lipid peroxidation, and lower antioxidant enzyme activities compared to other organs. Recent reports showed that hippocampal pyramidal neurons are more susceptible to oxidative stress induced damage compared to neurons at prefrontal cortex and cerebellum [8]. Administration of free radical quenchers like quercetin or antioxidant precursors such as N-acetyl cysteine has been reported to enhance cell viability in hypoxic stress [9]–[10].

Glutathione a tripeptide comprised of glutamate, cysteine and glycine, is a major antioxidant in the brain [11], with a concentration of approximately 2–3 mM. Glutathione is synthesized in cytosol by the consecutive action of the enzymes glutamate-cysteine ligase and glutathione synthetase which involve the utilization of ATP. Glutamate- cysteine ligase is the rate-limiting enzyme of GSH synthesis and is subjected to feedback inhibition by GSH [12]. Both enzymes are transcriptionally regulated by nuclear factor erythroid 2-related factor 2 (Nrf2), a redox-sensitive transcription factor member of the basic-leucine zipper family [13]. In response to oxidative stress, Nrf2 dissociates from its cytosolic inhibitor Keap1, translocates to the nucleus and binds to antioxidant-response elements (AREs) in the promoters of target genes. This leads to transcriptional induction of several cellular defense genes, including glutathione biosynthetic enzymes (glutathione cysteine ligase modifier subunit (GCLM) and catalytic subunit (GCLC) and GSH-dependent antioxidant enzymes (glutathione peroxidase 2, glutathione S-transferases and heme oxygenase-1) [14]. The Nrf2-mediated regulation of cellular antioxidant plays an important role in defense against oxidative stress [15]. Prophylactic Nrf2 activation by small molecules provide protection against a host of oxidative insults both *in vitro* as well as *in vivo*, including free radical donors and oxygen glucose deprivation (OGD), toxic levels of glutamate or N-methyl-D-aspartate (NMDA), neurotoxin or stroke-induced injury [16]. Exposure to hypobaric hypoxia depletes the neuronal glutathione in hippocampus [17]. Exogenous supplementation of GSH either through oral or intravenous route is hydrolyzed by γ-glutamyltranspeptidase and is rapidly eliminated within seven minutes from general circulation. Comford et al. showed that only 0.5% of radiolabeled GSH administered by intra-carotid injection was detectable in brain extracts [18]. Although there are reports describing the existence of GSH transporters, glutathione generally doesn't cross the blood-brain-barrier [19]. Hence, compounds modulating the GSH biosynthesis play a much significant role in providing protection against oxidative insult compared to exogenous supplementation of GSH. Since a batteries of free radicals scavenging enzyme system depend directly on availability of GSH for detoxification of ROS, molecules capable of modulating glutathione biosynthesis could potentially protect free radicals mediated neurodegenration under hypoxic condition.

The root extract of *Withania somnifera* is used as a popular herbal drug in Ayurvedic medicine, and has been used traditionally as a tonic and nootropic agent. It facilitate cognitive function and augment mental retention capacity following diabetes, Aβ and scopolamine induced memory loss [20]–[21]. It is also known to augment cholinergic activity in hippocampus [22]. Recent reports from our laboratory showed that withanolide enriched extract of *Withania somnifera* root ameliorates hypoxia induced memory impairment by modulating corticosterone level in brain through Nitric oxide cyclooxygenase prostaglandin pathway [17]. Methanolic extract of Withania root demonstrate profound association with neurite extension and dendritic arborisation [23]. Treatment with withanolide A (WL-A), a major active constituents isolated from *Withania somnifera* root predominantly induces axonal outgrowth in normal cortical neurons [24]. Supplementation of withanolide enriched extract of *Withania somnifera* root restored hypoxia induced depleted antioxidant glutathione level and free radical scavenging enzyme system in brain [17]. Since glutathione is the major antioxidant in brain, modulation of its biosynthesis by withanolide A under hypoxic condition could ameliorate oxidative stress induced neurodegeneration and consequent memory dysfunction. In the present study, we investigated the effect of withanolide A on hippocampal glutathione biosynthesis during hypoxic exposure and its correlation with hypoxia induced neurodegeneration and memory dysfunction. The study further explores the possible mechanism underlying withanolide A mediated modulation of glutathione system during exposure to hypobaric hypoxia.

## Materials and Methods

### Ethics Statement

All the protocols followed in this experiment were approved by the Institutional Committee for Animal Care and Use (ICACU), Defense Institute of Physiology and Allied Sciences, New Delhi (Permit Number: DIP-12-250) following the guidelines of “Committee for the Purpose of Control and Supervision of Experiments on Animals” Govt. of India. Utmost care was taken to minimize suffering of animal during sampling. Sacrifice of the animals were done under sodium pentabarbitol anaesthesia.

### Chemicals and reagents

Withanolide A (Cat # 74776; Purity ≥95%), corticosterone, buthionine sulfoximine (BSO) and metyrapone were procured from Sigma chemicals (Sigma-Aldrich, USA). Kits for estimation of glutathione reductase, glutathione peroxidase and superoxide dismutase activity were purchased from RANDOX (Randox laboratory, UK). Glutathione s transferase assay kit was procured from Caymen (Cayman, USA). All the primary and secondary antibodies used in the experiments were procured from abcam (abcam, USA). ATP chemiluminescence assay kit and EnzyChrom NADP+/NADPH Assay Kit was procured from Calbiochem (Calbiochem, San Diego, CA) and Bioassay System (Hayward, CA, USA) respectively. ABC staining kit for immunohistochemistry was purchased from the vectastain (Vector laboratory, USA). Superscript first strand cDNA synthesis kit and SYBR Green PCR Master Mix for real time analysis of GCLC and Nrf2 was purchased from Applied Biosystems (Applied Biosystems, Foster City, CA).

### Animals

Adult male Sprague Dawley rats weighing 240–250 g were taken and maintained at 12 h light-dark cycle (lights on from 8:00 AM–8:00 PM) in the animal house of the institute. Food pellets (Lipton Pvt. Ltd., India) and water was given *ad libitum*. The temperature and humidity of the animal house was maintained at 25±2°C and 55±5% respectively. All animal handling was performed between the time windows of 10.00 AM to 11.30 AM to avoid experimental deviations due to diurnal variations in corticosterone concentration.

### Hypoxic exposure

Animals were exposed to a simulated altitude of 7600 m (25,000 ft, 282 mm Hg) in a specially designed animal decompression chamber where altitude could be maintained by reducing the ambient barometric pressure. Periodic evaluation of fluctuation in oxygen level arising from fresh air flush into the chamber was done using an oxygen sensor. The temperature and humidity in the chamber were maintained precisely at 25±2°C and 55±5% respectively. The rate of ascent and descent to hypobaric conditions was maintained at 300 m/min as described previously [25]–[26]. The hypobaric hypoxic exposure was continuous for the stipulated period except for a 10–15 min interval each day for replenishment of food and water, drug administration and changing the cages housing the animals.

### Experimental design

**Figure 1:**
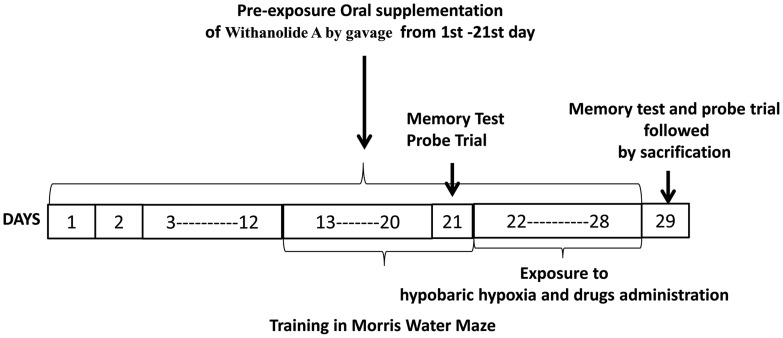
Showing the schedule of the training, probe trial and memory test in Morris Water Maze, supplementation of Withanolide A, administration of drugs and exposure to hypobaric hypoxia.

The study was performed in two phases. Phase I aimed at investigating the effect of withanolide A on glutathione level and GSH dependent free radical scavenging system in hippocampus following exposure to hypobaric hypoxia. Rats were screened using elevated plus maze and open field test to ensure that none of the animals selected for experimentation were having dysfunctions such as anxiety or locomotory problems. The selected rats were then divided into four groups randomly (n = 15/group) viz., normoxia, normoxia treated with withanolide A, hypoxia treated with vehicle (0.5% gum arabic solution) and hypoxic rats treated with withanolide A (Fig. 1). Alteration in level of reactive oxygen species, lipid peroxidation, GSH and activity of glutathione reductase, glutathione peroxidase, glutathione s transferase, superoxide dismutase and glutamyl cysteinyl ligase in hippocampal region were assessed. Changes in expression of glucocorticoid and mineralocorticoid receptor. corticosterone, ATP and NADPH level in hippocampal region was also estimated following hypoxic exposure.

Phase II study was conducted to explore the molecular mechanism underlying the modulatory effect of withanolide A on glutathione biosynthesis during exposure to hypobaric hypoxia and their effect on hypoxia induced neurodegeneration. Rats (n = 80) were divided into eight groups (n = 10/group) and drugs were administered as described in Table 1. The duration of hypoxic exposure was kept 7 days since neurodegeneration as well as the memory impairment was maximum on that day. Changes in apoptotic marker caspase 3 by immunohistochemistry and chromatin condensation by hoescht staining were evaluated in the CA3 region of hippocampus following hypoxic exposure and drugs administration.

**Table 1:** Showing schedules and doses of drug administered during exposure to hypobaric hypoxia.

Groups	Description	Hypobaric Hypoxia	Intervention administered	Duration of treatment	Dose and mode of administration of drugs	Nature of durgs
Group I (n = 10)	Normoxia + Withanolide A	No	Withanolide A#	28 days	10 µmol kg^−1^ day^−1^ (Oral)	
Group II (n = 10)	Hypoxia + Vehicle	Yes(07days)	None	05 days	None	
Group III (n = 10)	Hypoxia + Withanolide A	Yes(07days)	Withanolide A#	28 days	10 µmol kg^−1^ day^−1^ (Oral)	
Group IV (n = 10)	Normoxia + BSO	No	BSO	05 days	4 mM/Kg BW (i.p) (Ando et al., 2009)	Glutathione Synthesis Inhibitor
Group V (n = 10)	Hypoxia + BSO	Yes(07days)	BSO#	05 days	4 mM/KgBW	Glutathione Synthesis Inhibitor
Group VI (n = 10)	Hypoxia + Withanolide A+ BSO	Yes(07days)	BSO# Withanolide A#	05 days 28 days	4 mM/KgBW 10 µmol kg^−1^ day^−1^ (Oral)	
Group VII (n = 10)	Hypoxia + Withanolide A+ Corticosterone	Yes(07days)	Withanolide A Corticosterone#	28 days 05 days	10 µmol kg^−1^ day^−1^ (Oral) 40 mg/Kg BW (i.p) (Smith Swintosky et al., 1996)	
Group VIII (n = 10)	Hypoxia + Metyrapone	Yes(07days)	Metyrapone*	05 days	50 mg/Kg (i.p) (Baitharu et al., 2011)	Corticosterone Synthesis Inhibitor
Group IX (n = 10	Hypoxia + Corticosterone + Metyrapone	Yes(07days)	Corticosterone*Metyrapone*	05 days 05 days	40 mg/Kg 50 mg/Kg	Corticosterone Synthesis Inhibitor

* Indicate the administration of drug started from 3^rd^ day of hypoxic exposure.

# denotes the administration of Withanolide A started 21 days prior to hypoxic exposure and was continued during hypoxic exposure.

### Preparation of drug and pharmacological administration

Withanolide A was dissolved in 0.5% gum arabic solution and administrated to rats orally by gavage using feeding cannula at a dose of 10 µmol/kg^−^ BW (decided after dose optimization study). The Withanolide A feeding to rats was done for 21 days prior to and during exposure to hypobaric hypoxia for 7 days. Buthionine sulfoximine (4 mM/kg BW) was dissolved in phosphate buffer saline to a volume of 1 ml and administrered intraperitoneally (i.p) [27]. Metyrapone (50 mg/kg BW) was dissolved in the 40% polyethylene glycol (PEG) (w/v) in physiological saline. Metyrapone or an equivalent volume (1 ml) of the vehicle consisting of physiological saline and polyethylene glycol was injected intraperitoneally [28]. Corticosterone (40 mg/kg BW) was dissolved in peanut oil and injected subcutaneously [29] in a volume of 1 ml along with metyrapone during exposure to hypobaric hypoxia. Both the metyrapone as well as corticosterone administration was started from 3rd day and was continued till 7th day of hypoxic exposure. The drugs were administered once daily at 9:00 AM when decompression chamber was opened to replace food and water.

### Oxidative stress markers

On completion of the stipulated period of hypoxic exposure, rats were sacrificed and hippocampi were removed at 4–8°C in ice-cold 0.01 M phosphate buffer saline (PBS, pH 7.4). Tissue homogenates (10%) were prepared in 0.15 M KCl. The crude homogenates (250 µl) were taken for lipid peroxidation, GSH estimation and the remaining homogenates were centrifuged at 10,000 g for 30 min at 4–8°C. The supernatant was then collected and used for enzymatic estimations. The total protein content per 10 µl of each of the samples were estimated using bovine serum albumin as standard [30].

### Estimation of reactive oxygen species

Reactive oxygen species mainly hydrogen peroxide (H_2_O_2_) and peroxinitrite (ONOO ^−^) in the hippocampal tissue were estimated spectrofluorimetrically using 2,7-dichlorofluorescein-diacetate (DCFHDA) as suggested by LeBel et al. [31] and modified by Myhre et al.. [32]. In brief, hippocampal homogenate (10%) was prepared in ice cold 0.15 M KCl and 1.494 ml of 0.1 M PBS (pH 7.4) was added to 25 µl of the crude homogenate followed by addition of 6 µl of DCFHDA (1.25 mM) [25]. The sample was then incubated for 15 min at 37°C in dark and readings were taken at 488 nm excitation and 525 nm emission. The readings were expressed as fluorescent units per mg of protein and converted to percentage by taking normoxic value as 100%.

### Lipid peroxidation

Lipid peroxidation was measured by thiobarbituric acid test for malondialdehyde as per the method described by Das and Ratty [33] and modified by Colado et al.. [34]. Hippocampi were homogenized in 50 mM phosphate buffer, deproteinised with 40% trichloroacetic acid and 5 M hydrochloric acid. Thiobarbituric acid (2%) in 0.5 M sodium hydroxide was added to the deproteinised hippocampal sample. The reaction mixture was heated in a water bath at 90°C for 35 minutes and centrifuged at 12,000 g for 10 minutes. The pink chromogen formed was measured at 532 nm spectrophotometrically and expressed in mmol/mg protein. The results were then converted to percentage considering normoxic value as 100%.

### Reduced glutathione

The reduced gluathone in 10% hippocampal tissue homogenate was measured as per the protocol followed by Hissin and Hilf [35]. In brief, 250 µl of the crude homogenates were taken, to which equal volume of 10% metaphosphoric acid was added. The mixture was then centrifuged at 10,000 g for 30 minutes at 4°C. The supernatants obtained were used for the estimation of GSH by incubation with o-pthaldehyde. Readings were taken spectrofluorometrically at 350 nm excitation and 420 nm emission. The amount of GSH was calculated using a standard curve and expressed in mmol/mg of protein and converted to percentage taking normoxic value as 100%.

### Glutathione Reductase and Glutathione Peroxidase activity

Glutathione reductase activity was measured as per the method described by Pinto and Bartley [36] and the values obtained were expressed in mmol of NADPH oxidized/min/g tissue. The glutathione peroxidase (GPx) (EC 1.11.1.9) activity was measured using glutathione peroxidase assay kit and the results obtained were expressed in U/mg protein and converted to percentages taking normoxic value as 100%.

### Glutathione s transferase activity

The glutathione s transferase activity in the hippocampal tissue was estimated using glutathione s transferase activity assay kit as per the manufacturer protocol. Briefly, tissue was homogenized with ice cold 100 mM potassium phosphate buffer containing EDTA and centrifuged at 10,000 g for 15 minutes at 4°C. Supernatant was collected for assay. Optical density was measured spectrophotometrically (Molecular Devices, USA). The results obtained were expressed in U/mg protein and converted to percentages taking normoxic value as 100%.

### Super oxide dismutase activity

The superoxide dismutase activity in the hippocampal tissue was estimated using RANDOX kit (RANDOX Laboratory Ltd.). The activity of the enzymes was expressed as U/mg protein and converted to percentages taking normoxic value as 100%.

### ATP Level in hippocampus

The ATP content was determined using an ATP chemiluminescence assay kit (Calbiochem, San Diego, CA) as per the manufacturer's instructions. In brief, the tissue homogenate was treated with nuclear-releasing buffer for 5 min at room temperature with gentle shaking. To the tissue lysate, ATP monitoring enzyme was added and the luminescent reaction was immediately analyzed in a microplate reader (Spectra Max MII, Molecular Devices, Germany). The absolute ATP content was calculated by running an ATP standard curve with known ATP concentrations. Protein concentrations of samples were determined by Bradford assay (Bradford, 1976). The calculated total ATP concentration was expressed as nanomolar ATP/mg protein and converted to percentages taking normoxic value as 100%.

### Estimation of NADP+/NADPH level in Hippocampus

The NADP+/NADPH ratio was determined by using the EnzyChrom NADP^+^/NADPH Assay Kit (ECNP-100) procured from BioAssay Systems, (BioAssay Systems, Hayward, CA, USA). Briefly, Samples were homogenized with NADP^+^ extraction buffer for NADP^+^ determination and NADPH extraction buffer for NADPH determination separately. The tissue extracts was heated at 60°C for 5 min followed by addition of assay buffer and the opposite extraction buffer to neutralize the extracts. The mixture was spinned at 14,000 rpm for 5 min. Supernatant was used for NADP^+^/NADPH assays. Determination of both NADP^+^ and NADPH concentrations requires extractions from two separate samples. Calibration curve was prepared using NADP premix by mixing 1 mM standard and distilled water. Optical density (OD_0_) was read for time “zero” at 565 nm and OD_30_ after a 30 min incubation at room temperature. OD_0_ was subtracted from OD_30_ for the standard and sample wells and OD values were used to determine sample NADP+/NADPH concentration from the standard curve. The results thus obtained were converted to percentage considering normoxic values as 100%.

### Estimation of corticosterone level in hippocampus by High Performance Liquid Chromatography

Levels of corticosterone was estimated in hippocampal tissue using high performance liquid chromatography (Waters, Milford, MS, USA). The extraction of corticosterone from hippocampal tissue was done with diethyl ether [37]. The ether evaporated tissue samples were reconstituted with 250 µl of methanol. 10 µl of the reconstituted sample was injected with the help of an auto sampler (Waters) to the HPLC system and resolved using C18 RP column with acetonitrile: Water: Glacial acetic acid (35: 65∶05 v/v) as solvent phase in isocratic condition. The flow rate of the mobile phase was maintained at 1 ml/min and detection of corticosterone fraction was done at 254 nm with a UV detector. The pressure in the column was maintained at 1800 psi and the samples were run for 30 minutes. A standard plot was prepared using corticosterone standard and methanol in the range of 10–1000 ng/ml by serial dilution. The standards were tested individually at different concentrations to record detection limit, retention time and peak area. Concentration of corticosterone was calculated from a standard plot of peak area of corticosterone versus concentration of corticosterone.

### Determination of γ-GCL activity

**γ**-GCL activity was determined following the method described by Seelig et al.. [38]. Briefly, enzyme activity was determined at 37°C in reaction mixtures of 1.0 ml containing 100 mm Tris-HCl buffer (pH 8.2), 150 mm KCl, 5 mm ATP, 2 mm phosphoenolpyruvate, 10 mm glutamate, 10 mm **γ** -aminobutyrate, 20 mm MgCl_2_, 2 mm EDTA, 0.2 mm NADH, 17 µg pyruvate kinase, and 17 mg lactate dehydrogenase. The reaction was initiated by adding extract, and the rate of decrease in absorbance at 340 nm was monitored. Enzyme-specific activity was measured as micromoles of NADH oxidized per minute per milligram protein. The results thus obtained were converted to percentage considering normoxic values as 100%.

### Glucocorticoid receptor (GR), Mineralocorticoid receptor (MR), GCLC and Nrf2 in hippocampus by western blotting

The expression analysis of the proteins in the hippocampal region of the brain by western blotting was performed as described by Hota et al.. [25]. The hippocampi were dissected out at 4°C from the rat brain following decapitation and homogenized in ice-cold lysis buffer (0.01 M Tris–HCl, pH 7.6, 0.1 M NaCl, 0.1 M dithiothreitol, 1 mM EDTA, 0.1% NaN3, PMSF, Protease inhibitor cocktail). The homogenates were centrifuged at 10,000 g for 10 min at 4°C and the supernatants were used for protein expression analysis. SDS-PAGE (12%) was run in duplicates depending upon the molecular weight of the proteins of interest. Sample protein (50 mg) was resolved by SDS-PAGE and transferred to nitrocellulose membranes pre-soaked in transfer buffer (20% methanol, 0.3% Tris, 1.44% glycine in water) using a semidry transblot module (Bio-Rad). The transfer of the protein bands to the membrane was verified by Ponceau staining. The membranes were then blocked with 5% Blotto for 1 h, washed with PBST (0.01 M PBS, pH 7.4, 0.1% Tween 20) and probed overnight with polyclonal GR, MR, GCLC, GCLM and Nrf2 specific antibodies (abcam, USA). Subsequently, the membranes were washed with PBST thrice (10 min each) and were incubated with suitable secondary anti-IgG HRP conjugated antibody for 2–3 h. Chemiluminiscent peroxidase substrate kit was used to develop the membrane which were then stripped using stripping buffer (Bio-Rad) and probed for b-actin expression which was considered as loading control. The protein expression in each group was quantified by densitometric analysis.

### Real-time polymerase chain reaction (PCR) of Nrf2 and γ-GCLC mRNA

Total RNA was extracted from the hippocampal tissues using TRIzol Reagent (Invitrogen) and reverse transcribed using the Superscript First-Strand Synthesis System (Invitrogen). To quantify the gene expression levels in the samples, real-time polymerase chain reaction was performed on an ABI Prism 7700 Sequence Detection System using SYBR Green PCR Master Mix (Applied Biosystems, Foster City, CA) and primers specific for the catalytic subunits of glutamate-cysteine ligase (GCL; EC 6.3.2.2) of rats; i.e., GCLC (forward primer, 5′-CTCTGCCTATGTGGTATTTG-3′; reverse primer, 5′-TTGCTTGTAGTCAGGATGG-3′; amplicon size, 454 bp) and Nrf2 (forward primer, 5′ CGTGGTGGACTTCTC TGCTACGTG GTG 3′; reverse primer, 5′ GGTCGGCATGCATTTGACTTCACAGTC 3′; amplicon size, 352 bp) and primers for β-actin to normalize the amount of mRNA in the samples (forward primer, 5′-TCTTCCAGCCTTCCTTCC-3′; reverse primer, 5′-TAGAGCCACCAATCCACAC- 3′; amplicon size, 252 bp). The annealing temperature and the primer concentrations were optimized for amplification efficiency after validation of the dissociation curves and satisfactory separation of the PCR products on a 1.5% agarose gel. The optimal thermal cycle protocol for all the samples began with 10-min denaturation at 95°C, followed by 40 cycles of 95°C for 15 s, 62°C for 30 s, and 72°C for 45 s. The concentrations of the primers used for GCLC, Nrf2 and β-actin were 200, 260, and 200 µM, respectively. The relative amounts of mRNA for GCLC and Nrf2 in the drug treated groups versus the vehicle treated hypoxic and normoxic group were calculated as the relative expression ratios in comparison with β-actin and expressd in fold change.

### Immunohistochemistry of caspase 3 in CA3 region of hippocampus

In brief, sections were washed in 0.1 M Phosphate Buffer Saline (pH 7.4) for 30 min, and treated with 0.3% hydrogen peroxide for 30 min to inhibit endogenous peroxidase activity. Sections were washed in 0.1 M PBS, permeabilized with 0.25% Triton X-100 and blocked with 1.5% goat serum for 3 h at room temperature. They were then incubated with rabbit anti-Caspase 3 (1∶200) antibodies for 48 hours at 4°C. The sections were then washed and incubated with biotinylated goat anti-rabbit immunoglobulin (1∶100) for 3 h at room temperature, washed with 0.1 M PBS (pH 7.4) and incubated with avidin-biotin complex tagged with horse redox peroxidase enzyme prior to development with 3, 3′-diaminobenzidine (ABC kit, SantaCruz). Digital images were acquired using a light microscope (Olympus, Model BX 51, Japan) and the immunoreactive neurons for Caspase 3 were counted by Image Pro-Plus 5.1 software in six random fields of 0.1 mm^2^.

### Chromatin condensation by Hoechst staining

Chromatin condensation, which is an indicator of apoptosis, was studied by Hoechst 33342 staining. Hippocampal sections (15 µm thickness) were permeabilized in 0.1% triton and stained with Hoechst 33342 (10 mg/ml) for 30 min in the dark. The stained 6.04-mm sections of bregma were visualized using a blue filter in an Olympus BX-51 fluorescent microscope, and the brightly fluorescing cells were scored qualitatively.

### Memory assessment by Morris Water Maze

Morris Water Maze was used to investigate the spatial reference memory of rodents [39]. An overhead camera and computer assisted tracking system with videomax software (Columbus Instruments, USA) was used to record the position of the rat in the maze. During reference memory task the rats were trained for a period of 8 days (sessions) followed by a probe trial on the ninth day. The platform position was kept fixed in one position throughout the training period. The rats were released randomly choosing any of the four quadrants as starting position and the starting position was changed in each release. The order of the starting position varied in every trial and no given sequence was repeated. The number of crossing over at the original platform position and the time (s) spent in the target quadrant were calculated. The rats were then exposed to hypobaric hypoxia for 7 days following which the reference memory was tested by a probe trial for 60 s and a single trial for memory test to locate the submerged platform. The amount of platform crossings and the time spent in the target quadrant during the probe trial and the latency as well as path length during the single trial was considered as measures for assessment of memory.

### Statistical Analysis

Probe trial task in Morris Water Maze after exposure to hypoxia was analyzed using one-way Analysis of variance. Mean of latencies and pathlength for reference memory testing after exposure to hypobaric hypoxia was analyzed in similar manner since one trial was given for each task. The results of oxidative stress markers and other biochemical parameters are representations of six individual observations and presented as means ± SEM unless otherwise mentioned. Statistical analysis for multiple comparisons was done between normoxic group, hypoxic group and hypoxia with drug treated groups using one and two-way ANOVA wherever applicable. The post hoc analysis was done by Newman– Keul's test in all experimental groups wherever appropriate. Difference below or equal to the probability level (p≤0.05) was considered statistically significant.

## Results

### Withanolide A reduces oxidative stress in hippocampal region of the brain during exposure to hypobaric hypoxia

**Figure 2:**
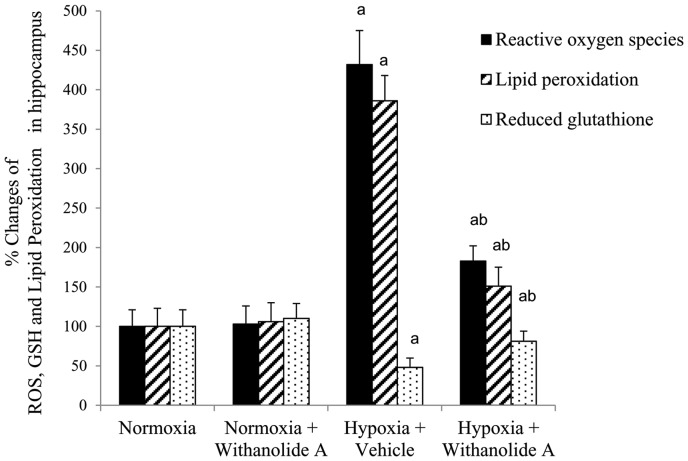
Effect of Withanolide A on oxidative stress markers. Administration of Withanolide A decreases the hypoxia induced elevated level of reactive oxygen species, lipid peroxidation and GSH in hippocampus. Data expressed as percentage change taking normoxic value as 100% and represents Mean ± SEM. ‘a’ denotes p≤0.05 vs. when compared to normoxic group and ‘b’ denotes p≤0.05 vs. when compared to 7 days hypoxic group treated with vehicle only.

Exposure to hypobaric hypoxia significantly elevated the level of reactive oxygen species generation (F (3, 20) = 22.3, p≤0.05) and lipid peroxidation (F (3, 20) = 18.1, p≤0.05) along with significant reduction in GSH level (F (3, 20) = 21.8, p≤0.05) in hippocampus compared to normoxic group. Supplementation of withanolide A 21 days before and during exposure to hypobaric hypoxia for 7 days significantly decreased the free radicals level and lipid peroxidation and significantly increased GSH level in hippocampus compared to hypoxic vehicle treated group as shown in Fig. 2.

### Withanolide A modulates GSH dependent free radicals scavenging system in hippocampus during hypobaric hypoxia

**Figure 3:**
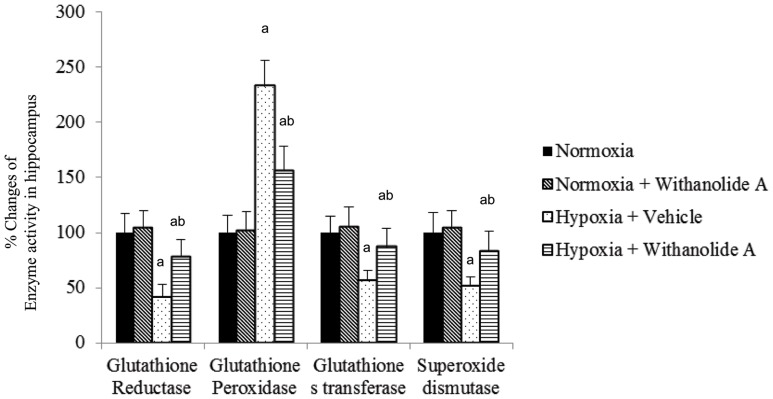
Effect of Withanolide A on free radical scavenging enzyme system. Withanolide A administration during hypoxic exposure increases hypoxia induced decreased activity of glutathione reductase, glutathione peroxidase, glutathione s transferase and superoxide dismutase in hippocampus. Data expressed as percentage change taking normoxic value as 100% and represents Mean ± SEM. ‘a’ denotes p≤0.05 vs. when compared to normoxic group and ‘b’ denotes p≤0.05 vs. when compared to 7 days hypoxic group treated with vehicle only.

The activity of glutathione reductase (F (3, 20) = 14.2, p≤0.05), glutathione s transferase (F (3, 20) = 11.3, p≤0.05), and superoxide dismutase (F (3, 20) = 09.3, p≤0.05) was significantly decreased with concomittent increased glutathione peroxidase activity following exposure to hypobaric hypoxia compared to normoxic group. Supplementation of withanolide A 21 days before and 7 days during exposure to hypobaric hypoxia significantly increased the activity of glutathione reductase, glutathione s transferase and superoxide dismutase with significant decrease in GPx activity compared to hypoxic group treated with vehicle only (Fig. 3).

### Administration of Withanolide A during expoaure to hypobaric hypoxia alters ATP, NADPH level and GCLC activity in hippocampus

**Figure 4:**
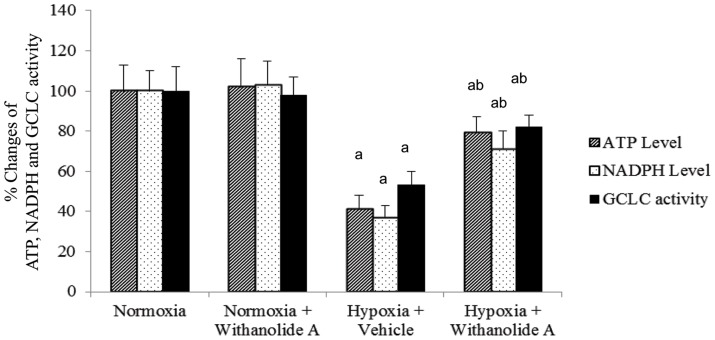
Restoration of ATP, NADPH and γ-glutamylcysteinyl ligase catalytic subunit activity in hippocampus following Withanolide A during hypoxia. Hypoxic exposure for 7 days decreases the hippocampal ATP, NADPH and γ-glutamylcysteinyl ligase catalytic subunit activity while administration of Withanolide A increases the ATP, NADPH and γ-glutamylcysteinyl ligase catalytic subunit activity in hippocampus. Data expressed as percentage change taking normoxic value as 100% and represents Mean ± SEM. ‘a’ denotes p≤0.05 vs. when compared to normoxic group and ‘b’ denotes p≤0.05 vs. when compared to 7 days hypoxic group treated with vehicle only.

There was significant decrease of ATP level (F (3, 20) = 16.1, p≤0.05), NADPH level (F (3, 20) = 12.3, p≤0.05) and GCLC activity (F (3, 20) = 14.7, p≤0.05) in hippocampal region following exposure to hypobaric hypoxia compared to normoxic group. Admnistration of withanolide A 21 days prior to and during exposure to hypobaric hypoxia for 7 days significantly increased the ATP, NADPH and GCLC activity compared to hypoxic group treated with vehicle only as shown in Fig. 4.

### Withanolide A modulates hippocampal corticosterone and its receptors during exposure to hypobaric hypoxia

Exposure to hypobaric hypoxia for 7 days significantly (F (3, 20) = 26.5, p≤0.05) elevated the corticosterone level, glucocorticoid F (3, 20) = 15.6, p≤0.05) and mineralocorticoid receptor F (3, 20) = 21.4, p≤0.05) in hippocampus compared to normoxic group. Significant decrease in corticosterone level and glucocorticoid receptor in hippocampus was observed when administered with withanolide A 21 days prior to and during exposure to hypobaric hypoxia for 7 days compared to hypoxic group treated with vehicle only while no difference was noted in the mineralocorticoid receptor expression in hippocampus (Fig. S1 and S2).

### Withanolide A provide neuroprotection during hypoxic exposure by modulating corticosterone level in hippocampus

Exposure to hypobaric hypoxia significantly (F (4, 26) = 13.33, p≤0.05) increased the number of pycknotic cells in hippocampal region compared to normoxic group. Supplementation of withanolide A during exposure to hypobaric hypoxia significantly decreased the number of pycknotic cells in the CA3 region of hippocampus compared to hypoxic group treated with vehicle only. Administration of Withanolide A as well as metyrapone during exposure to hypobaric hypoxia significantly decreased the number of pycknotic neurons in the CA3 region of hippocampus compared to Hypoxic group treated with vehicle only as shown in Fig. S3.

### Withanolide A restores hypobaric hypoxia induced memory impairment in rats

**Figure 5:**
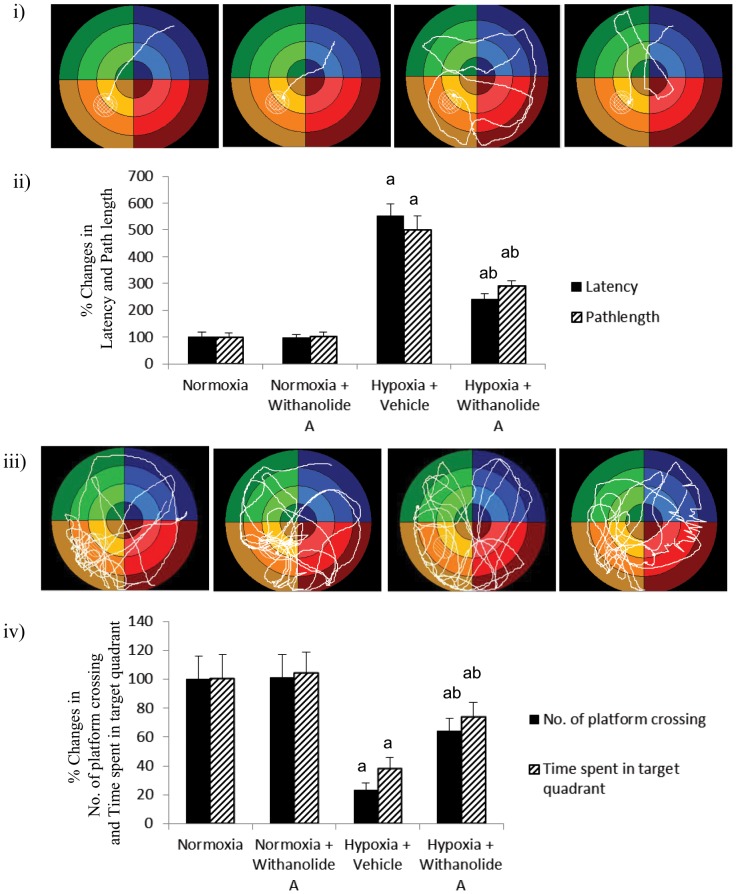
Amelioration of spatial memory function following Withanolide A administration during hypoxic exposure. Exposure to hypobaric hypoxia increased (i) path length and (ii) latency during spatial memory test but decreased (iii) number of platform crossing and (iv) time spent in target quadrant during probe trial when was reversed following withanolide A supplementation before and during exposure to hypobaric hypoxia. Data expressed as percentage change taking normoxic value as 100% and represents Mean ± SEM. ‘a’ denotes p≤0.05 vs. when compared to normoxic group and ‘b’ denotes p≤0.05 vs. when compared to 7 days hypoxic group treated with vehicle only.

Exposure to hypobaric hypoxia significantly increased the latency as well as pathlength in Morris Water Maze during memory test compared to normoxic group. Supplementation of withanolide A during exposure to hypobaric hypoxia significantly decreased the latency (F (3, 56) = 9.13, p≤0.05) and pathlength (F (3, 56) = 8.71, p≤0.05) during memory test compared to hypoxic group treated with vehicle only as shown in Fig. 5 i and ii. On the other hand, there was significant decrease in number of platform crossing (F (3, 56) = 11.13, p≤0.05) as well as time spent in target quadrant during probe trial (F (3, 56) = 8.13, p≤0.05) following exposure to hypobaric hypoxia compared to normoxic group. Administration of withanolide A 21 days prior to and during exposure to hypobaric hypoxia for 7 days significantly increased the number platform crossing and time spent in target quadrant during probe trial compared to hypoxic group trated with vehicle only (Fig. 5 iii and iv).

### Withanolide A reverses neuronal apoptosis in CA3 region of hippocampus following hypoxic exposure and depletion of GSH

**Figure 6:**
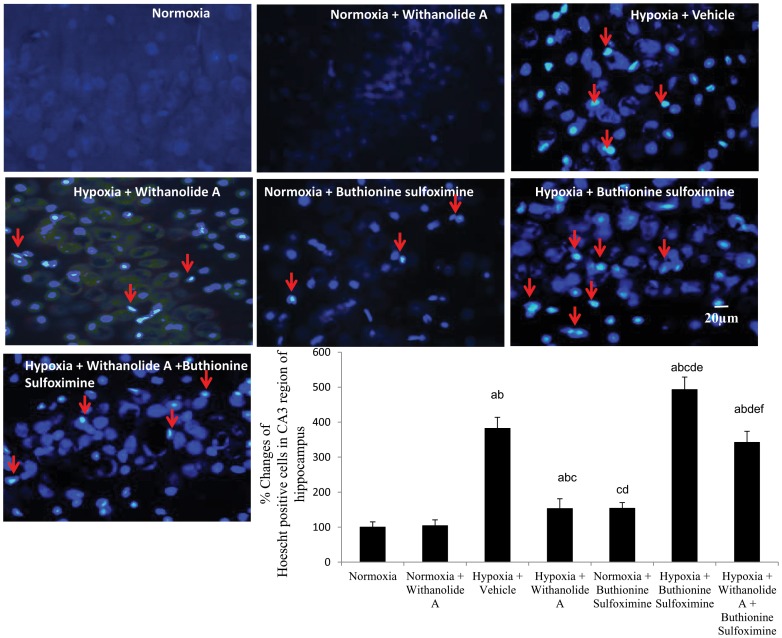
Modulation of hippocampal endogenous glutathione level by Withanolide A reduces hypoxia induced neurodegeneration. Withanolide A effectively decreases the number of degenerating neurons caused by hypoxic exposure. However, depletion of glutathione using buthionine sulfoximine during hypoxic exposure increases the number degenerating cells in hippocampus. Co-administration of withanolide A alongwith buthionine sulfoximine attenuates the neuroprotective effect of withanolide A during hypoxia. Data expressed as percentage change taking normoxic value as 100% and represents Mean ± SEM. ‘a’ denotes p≤0.05 vs. when compared to normoxic group. ‘b’ denotes p≤0.05 vs. when compared to normoxia + withanolide A group and ‘c’ denotes p≤0.05 vs. when compared to hypoxia + vehicle group, ‘d’ denotes p≤0.05 vs. when compared to hypobaric hypoxia + withanolide A, ‘e’ denotes p≤0.05 vs. when compared to normoxia + buthionine sulfoximine, and ‘f’ denotes p≤0.05 vs. when compared to hypobaric hypoxia + buthionine sulfoximine.

**Figure 7:**
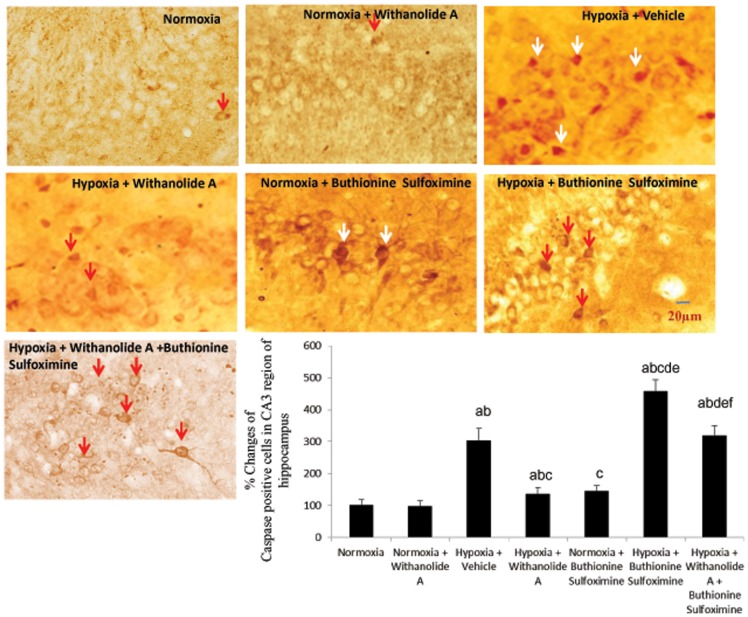
Withanolide A mediated restoration of endogenous glutathione level reverses hypoxia induced neuronal apoptosis in hippocampus. Administration of withanolide A prior to and during hypoxic exposure decreases number of apoptotic cells in CA3 region of hippocampus while glutathione depletion using buthionine sulfoximine elevates hypoxic neuronal apoptosis. Co-administration of withanolide A alongwith buthionine sulfoximine during hypoxic exposure enhances hypoxia induced apoptotic cells attenuating the nuroprotective effect of withanolide A. Data expressed as percentage change taking normoxic value as 100% and represents Mean ± SEM ‘a’ denotes p≤0.05 vs. when compared to normoxic group. ‘b’ denotes p≤0.05 vs. when compared to normoxia + withanolide A group and ‘c’ denotes p≤0.05 vs. when compared to hypoxia + vehicle group, ‘d’ denotes p≤0.05 vs. when compared to hypobaric hypoxia + withanolide A, ‘e’ denotes p≤0.05 vs. when compared to normoxia + buthionine sulfoximine, and ‘f” denotes p≤0.05 vs. when compared to hypobaric hypoxia + buthionine sulfoximine.

Exposure to hypobaric hypoxia significantly (F (6, 36) = 11.33, p≤0.05) increased the number of apoptotic caspase 3 positive cells and hoescht positive cells (F (6, 36) = 21.48, p≤0.05) in hippocampal region compared to normoxic group. Administration of buthionine sulfoximine during hypoxic exposure significantly increased the number of apoptotic cells and hoesct positive cells in hippocampus compared to hypoxic group treated with vehicle only. Though supplementation of withanolide A during exposure to hypobaric hypoxia significantly decreased the number of caspase positive cells as well as hoescht positive cells in the CA3 region of hippocampus compared to hypoxic group treated with vehicle only, combined administration of buthionine sulfoximine along with Withanolide A during hypoxic exposure significantly elevated the number of apoptotic cells in CA3 region of hippocampus compared to hypoxic group treated with Withanolide A only and vehicle treated group (Fig. 6 and 7 i and ii). Double labelling of apoptotic marker caspase 3 and neuronal marker NeuN further indicate the death of neurons in CA3 region hippocampus (Fig. S4).

### Withanolide A maintains redox homeostasis by modulating glutathione biosynthesis in hippocampus through regulation of Nrf2 pathway and corticosterone signaling during hypoxic exposure

**Figure 8:**
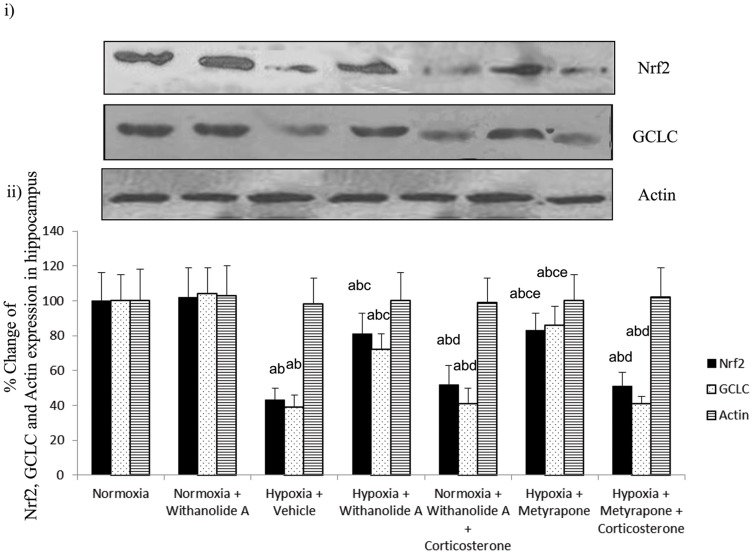
Withanolide A mediated elevation of hippocampal glutathione during hypoxia is corticosterone dependent. Withanolide A administration during hypoxic exposure upregulates Nrf2 and GCLC expression in hippocampus. Administration of Withanolide A alongwith exogenous corticosterone supplementation to the normoxic group decrease the Nrf2 as well as GCLC expression while inhibition of corticosterone synthesis using metyrapone reverses hypoxia induced downregulation of both Nrf2 and GCLC level. β-actin was used as a loading control. Data expressed as percentage change taking normoxic value as 100% and represents Mean ± SEM. ‘a’ denotes p≤0.05 vs. when compared to normoxic group. ‘b’ denotes p≤0.05 vs. when compared to normoxia + withanolide A group and ‘c’ denotes p≤0.05 vs. when compared to hypoxia + vehicle group, ‘d’ denotes p≤0.05 vs. when compared to hypobaric hypoxia + withanolide A, ‘e’ denotes p≤0.05 vs. when compared to hypobaric hypoxia + withanolide A + corticosterone and ‘f” denotes p≤0.05 vs. when compared to hypobaric hypoxia + Metyrapone.

There was significant decrease in the expression of Nrf2 (F (6, 22) = 17.36, p≤0.05) and γ-GCLC (F (6, 22) = 21.13, p≤0.05) in hippocampus following exposure to hypobaric hypoxia for 7 days compared to normoxic group. Supplementation of withanolide A 21 days before and during exposure to hypoxic exposure significantly increased Nrf2 and γ-GCLC expression in hippocampus compared to hypoxic group treated with vehicle only. However, administration of corticosterone along with Withanolide Aduring exposure to hypobaric hypoxia significantly reduced the expression of Nrf2 as well as γ-GCLC in hippocampal region compared to hypoxic group treated with withanolide A only. Further, administration of metyrapone during hypoxic exposure significantly increased the Nrf2 and γ-GCLC expression in hippocampus compared to withanolide A and corticosterone administered group. On the contrary, exogenous supplementation of corticosterone along with metyrapone significantly reduced the expression of Nrf2 and γ-GCLC in hippocampal region of brain compared to only metyrapone treated group as shown in Fig. 8 i and ii.

### Withanolide A mediated transcriptional regulation of Nrf2 and γ-GCLC during hypoxic exposure is corticosterone dependent

**Figure 9:**
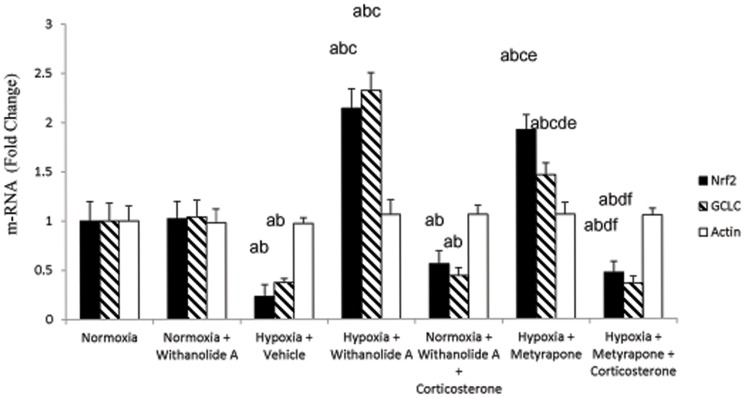
Withanolide A mediated transcriptional regulation of Nrf2 and GCLC expression depend on corticosterone signaling in hippocampus. Withanolide A administration during hypoxic exposure upregulates Nrf2 and GCLC expression in hippocampus. Administration of Withanolide A alongwith exogenous corticosterone supplementation to the normoxic group decrease the Nrf2 as well as GCLC expression while inhibition of corticosterone synthesis using metyrapone during hypoxia reverses hypoxia induced downregulation of both Nrf2 and GCLC level. β-actin was used as a loading control. Data expressed as percentage change taking normoxic value as 100% and represents Mean ± SEM. ‘a’ denotes p≤0.05 vs. when compared to normoxic group. ‘b’ denotes p≤0.05 vs. when compared to normoxia + withanolide A group and ‘c’ denotes p≤0.05 vs. when compared to hypoxia + vehicle group, ‘d’ denotes p≤0.05 vs. when compared to hypobaric hypoxia + withanolide A, ‘e’ denotes p≤0.05 vs. when compared to hypobaric hypoxia + withanolide A + corticosterone and ‘f” denotes p≤0.05 vs. when compared to hypobaric hypoxia + metyrapone.

Exposure to hypobaric hypoxia for 7 days significantly decreased the Nrf2 (F (6, 22) = 22.13, p≤0.05) and γ-GCLC m-RNA level (F (6, 22) = 17.43, p≤0.05) in hippocampus compared to normoxic group. Supplementation of withanolide A 21 days prior to and during exposure to hypoxic exposure significantly upregulated Nrf2 and γ-GCLC expression in hippocampus compared to hypoxic group treated with vehicle only. However, administration of corticosterone along with withanolide A during exposure to hypobaric hypoxia significantly reduced the expression of Nrf2 as well as γ-GCLC m-RNA level in hippocampal region compared to only withanolide A treated hypoxic group. Further, administration of metyrapone during hypoxic exposure transcriptionally upregulted the Nrf2 and γ-GCLC expression in hippocampus significantly compared to withanolide A and corticosterone administered group. On the contrary, exogenous supplementation of corticosterone along with metyrapone significantly reduced the expression of Nrf2 and GCLC m-RNA level in hippocampal region of brain compared to only metyrapone treated group (Fig. 9).

## Discussion

Hypobaric hypoxia induced prolonged elevation of corticosterone in hippocampus have been shown to cause enhanced oxidative stress, neurodegeneration and impairment of memory consolidation and retrieval. Inhibition of corticosterone synthesis or blockade of glucocorticoid receptor during hypoxic exposure reduced neurodegeneration and ameliorated memory impairment [28], [40]. However, use of synthetic inhibitors as prophylactic to prevent high altitude maladies may not be preferable because of their possible negative side effects. Recent studies from our laboratory demonstrate the prophylactic efficacy of withanolide enriched extract of *Withania somnifera* root in preventing hypoxia induced memory dysfunction by modulating corticosterone secretion and GSH level in hippocampal region of brain [17]. However, mechanism underlying such modulatory effect of withanolides on glutathione biosynthesis under hypoxic condition remains unexplored. Present investigation demonstrate that withanolide A causes augmented synthesis of glutathione in hippocampal neurons by upregulating key regulating enzyme for glutathione biosynthesis γ-glutamyl cysteinyl ligase and Nrf2 and attenuate hypoxia induced hippocampal neurodegeneration. The study further indicate that glucocorticoid signaling play a pivotal role in modulating glutathione level in neuron by regulating expression of Nrf2 and GCLC during hypoxia.

Glutathione is the most abundant thiol-containing molecule and is crucial for neuroprotection in the brain which non-enzymatically reacts with superoxide [41], NO [42], ONOO− and hydroxyl radicals [43]. It is the major redox buffer that maintains intracellular redox homeostasis. Under conditions of oxidative stress, GSH can lead to reversible formation of mixed disulfides between protein thiol groups through S-glutathionylation, a process critical for preventing irreversible oxidation of proteins [44]. Thus, GSH modulates a variety of protein functions via S- glutathionylation. While cysteine itself has neurotoxic effects mediated by free radical generation, increasing extracellular glutamate and triggering overactivation of N-methyl-D-aspartate (NMDA) receptors [45], GSH is a non-toxic cysteine storage form with 10–100 times higher concentrations in mammalian tissues than cysteine [46]. Further, GSH can serve as a neuromodulator that bind to NMDA receptor via its γ-glutamyl moiety and is known to exert dual (agonistic/antagonistic) actions on neuronal responses [45]. Keeping in mind the neuroprotective effects exerted by GSH in neuronal system, it is expected that molecules modulating GSH synthesis could be of potent therapeutics importance to cure neurodegenerative disorders.

Oxygen scarcity causes impairment of electron transport chain in mitochondria owing to its pivotal role as electron sink. Incomplete reduction of oxygen in hypoxic condition results in elevated production of superoxide and hydroxyl radicals. Exposure to hypobaric hypoxia induces oxidative stress in brain [5], [28]. Corroborating with previous findings, present study document an elevated level of free radicals and consequent lipid peroxidation following exposure to hypobaric hypoxia for 7 days along with a reduced level of endogenous antioxidant glutathione in hippocampus. The observed decrease in glutamylcysteinyl ligase activity, the key regulatory enzyme for gluatathione biosynthesis further support the decreased level of glutathione in hippocampus under hypoxic condition. Administration of withanolide A before and during exposure to hypobaric hypoxia decreased the free radical level which further diminished the incidence of lipid peroxidation. Similar reports on several other stresses like diabetes and chronic food shock showed that *Withania somnifera* root extract administration during stress exposure increase GSH level, reduce reactive oxygen species generation and lipid peroxidation [47]–[48]. The anti-oxidative effect of *Withania somnifera* root extract could be attributed to the rich content of withanolides, flavonoids and other components with strong antioxidant potential [49]. Interestingly, withanolide A induced augmentation of endogenous antioxidant GSH level in hippocampus point towards the efficacy of plant components in modulating glutathione biosynthesis or its stabilization under hypoxic condition [50]. Increased GCLC activity following supplementation of withanolide A support the involvement of withanolide A in modulating glutathione biosynthesis under hypoxic condition. Similar modulation of glutathione synthesis and upregulation of GCLC activity by several molecules like adrenomedullin, flavanoids like butein and phloretin following exposure to stressors causing oxidative load have been shown to provide augmented neuroprotection [51]–[52].

The brain contains high level of glutathione peroxidase which requires GSH for reduction of H_2_O_2_ and other peroxides [53], [4]. In the present study, decreased glutathione reductase activity with concomittant increase in activity of glutathione peroxidase following exposure to hypobaric hypoxia causes elevated accumulation of oxidized glutathione in hippocampus. Regeneration of oxidized form of glutathione to its reduced form depends on availability of NADPH and ATP in neuronal system. On the other hand, exposure to hypobaric hypoxia decreased the ATP and NADPH level in hippocampus which could be a result of impaired mitochondrial functioning on account of reduced availability of oxygen and malfunctioning of the pentose phosphate pathway that generate NADPH and/or overutilization of NADPH under hypoxic condition [26]. Winterbourn et al.., (1994) reported similar decreased level of ATP and NADPH under ischemic and hypoxic condition in brain and other tissues [41], [28]. Since synthesis of GSH is a ATP dependent process, decreased ATP level in hippocampus during hypoxic exposure could substantially influence the GSH level in hippocampus. Thus, it could be inferred that compromised glutathione biosynthesis and reduced availalbility of ATP and NADPH in hippocampal neuronal cells following exposure to hypobaric hypoxia impairs regeneration of glutathione from its oxidized state. Restoration of ATP and NADPH level, GCLC activity and augmented glutathione reductase activity following administration of withanolide A indicate the modulatory effect of withanolide A on glutathione biosynthesis and maintenance of neuronal redox potential in hippocampus.

Present study showed decreased activity of glutathione s transferase and superoxide dismutase activity in hippocampus following exposure to hypobaric hypoxia which was restored on supplementation of withanolide A. Elevation in glutathione s transferase and superoxide dismutase activity under hypoxic condition further supports the efficacy of withanolide A in modulating the free radicals scavenging enzyme system by maintaining neuronal GSH level. Similar elevated glutathione s transferase and superoxide dismutase activity following administration of *Withania somnifera* root extract and decrease oxidative stress induced neurodegeneration have been observed in restraint, ischemia and other moderate stress model [15], [54].

Nitric oxide (NO) being a diffusible retrograde neurotransmitter and signaling molecule exerts multipronged effect on neuronal survivability through formation of peroxynitrite [55] and activation of cGMP cascade [56]. Previous report from our laboratory demonstrate that exposure to hypobaric hypoxia elevate nitric oxide level in hippocampus [5]. Nitric oxide can directly stimulate augmented synthesis and secretion of corticosterone by NO-COX-Prostaglandin pathway and enhance hypoxia induced neurodegeneration [17]. Since GSH serves as an endogenous NO reservoir by forming S-nitrosoglutathione (GSNO) [57], withanolide A mediated elevated synthesis of GSH can modulate the nitric oxide level in hippocampus during hypoxic exposure through formation of nitrosoglutathione, thereby attenuating the toxic effect nitric oxide. Studies showed similar protective effect of GSNO in the brain under oxidative stress conditions by regulating NO release and exerts different biological effects [58]. However, further studies are needed to unfold effect of GSNO in high altitude hypoxic condition.

Reduced glutathione regulates both apoptotic and necrotic cell death by modulating the expression/activity of caspases and other signaling molecules [59]. Depletion of GSH during stress enhance oxidative insult and causes neurodegeneration. Maiti et al.., reported that loss of memory following exposure to hypobaric hypoxia occurs due to neuronal apoptosis in hippocampal region of the brain [60]. Present study showed that inhibition of key regulatory enzyme for glutathione biosynthesis GCLC using buthionine sulfoximine (BSO) during exposure to hypobaric hypoxia enhanced the hypoxia induced neurodegeneration as evident from elevated caspase positive and hoescht positive cells in the CA3 region of hippocampus. Similar studies showed that reduction of the brain glutathione content by buthionine sulfoximine (BSO), a specific inhibitor of GCLC enhances the toxic effects associated with elevated production of reactive oxygen species under ischemic condition [61]–[62] or treatment with 6-hydroxydopamine [63]. Decreased hoescht and caspase positive cells in CA3 region of hippocampus when administered with withanolide A indicate its neuroprotective effect under hypoxic condition. Interestingly, administration of buthionine sulfoximine along with withanolide A blunted its neuroprotective effect indicating the importance of glutathione biosynthesis in Withanolide A mediated neuroprotection. Similar studies also showed that GSH depletion in brain by treatment with buthionine sulfoximine leads to increased production of superoxide, hydroxyl radicals and H_2_O_2_ [64]. Decreased intracellular GSH on buthionine sulfoximine treatment worsen oxidative damage in hippocampus, while increased intracellular GSH by N-acetylcysteine (NAC) treatment ameliorated this damage [65], [9]. Thus, the study supports the fact that intracellular GSH pool is important for limiting oxidative stress induced neuronal injury.

The key regulatory enzyme for glutathione biosynthesis γ-glutamylcysteinyl ligase is regulated by various transcription factors and environmental stimuli. Nuclear factor (erythroid-derived 2)-like 2 (Nrf2) is one of the major transcription factor that maintain the redox homeostasis in neuronal system. In the present study, prolonged exposure to hypobaric hypoxia down regulates the antioxidant regulatory transcription factor Nrf2 in the brain. Nrf2 in turn can down regulate the key regulatory enzyme GCLC for glutathione biosynthesis. In the present study, supplementation of withanolide A reversed hypoxia induced down regulation of Nrf2 in the hippocampal region of the brain with concomitant increased expression of GCLC. On the other hand, supplementation of corticosterone during exposure to hypobaric hypoxia down regulated the Nrf2 and GCLC in hippocampus. Interestingly, inhibition of corticosterone synthesis during hypoxic exposure using metyrapone upregulated Nrf2 as well as GCLC in hippocampus suggesting the regulatory role of corticosterone in Nrf2 mediated induction of GCLC expression. Further, administration of withanolide A along with exogenous supplementation of corticosterone blunted upregulation of Nrf2 and GCLC in hippocampal region indicating that Withanolide A upregulate Nrf2 and GCLC by decreasing the corticosterone level in hippocampus. Baitharu et al. showed that administration of withanolide A enriched extract of *withania somnifera* root extract during hypoxic exposure decreased the corticosterone level as well as the glucocorticoid receptor expression in hippocampus [66]. Supporting the present findings, similar studies by Kratschmar et al. (2012) demonstrate that glucocorticoids suppress cellular antioxidant defence capacity by impairing Nrf2-dependent antioxidant response [67]. Furthermore, combined administration metyrapone and corticosterone nullified the upregulation of Nrf2 as well as GCLC confirming the role of corticosterone or its receptor in regulating Nrf2 expression under hypoxic condition. While exposure to hypobaric hypoxia increased corticosterone level in hippocampus, activation of glucocorticoid receptors by corticosterone can supress Nrf2 expression resulting in decreased expression of GCLC. Supporting our findings, studies by Ki et al.. showed that activated glucocorticoid receptor modulates Nrf2 signaling and alters of Nrf2 target genes expression in brain through binding of glucocorticoid receptor to its glucocorticoid response element [68]. However, exact mechanisms involved in regulation of Nrf2 by glucocorticoid receptor in hypoxia need further investigation.

### Conclusion

**Figure 10:**
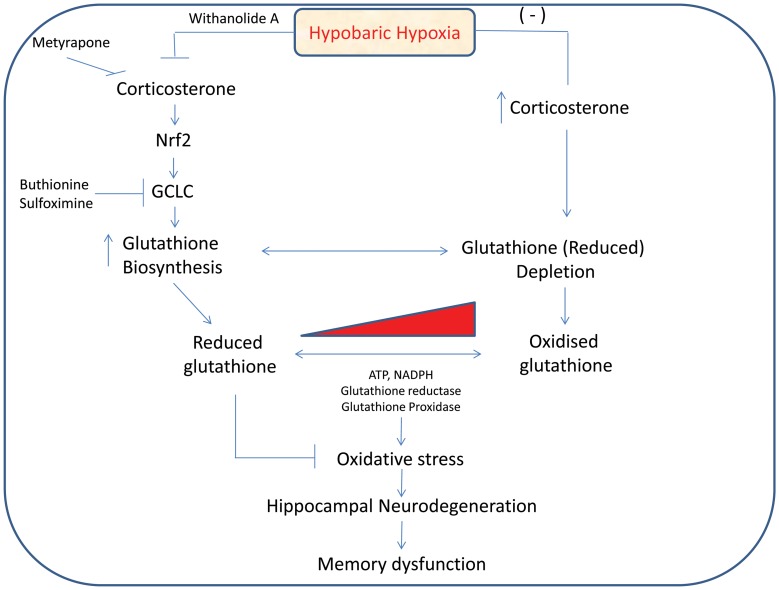
Schematic diagram showing Withanolide A mediated modulation of glutathione biosynthesis and neuroprotection during hypobaric hypoxia.

Present study demonstrate that in addition to its strong antioxidant property, withanolide A provide augmented neuroprotection by modulation of endogenous glutathione level in hippocampus during exposure to hypobaric hypoxia. Withanolide A increases glutathione biosynthesis in neuronal cells by upregulating GCLC level through Nrf2 pathway in a corticosterone dependenet manner (Figure 10). Since exogenous supplementation of GSH is not effective, modulation of glutathione biosynthesis by withanolide A could be of much therapeutic interest and can be used as a prophylactic to prevent/cure neurodegenerative disorders invoked by elevated oxidative insults in hypobaric hypoxia and other similar pathological condition.

### Supporting Information

Exposure to hypobaric hypoxia elevates corticosterone level in hippocampal tissue while withanolide A administration attenuate cortcosterone elevation and maintain it in optimal level as shown Fig. S1. Withanolide A administration during hypoxic exposure decreases glucocorticoid receptor and increases mineralocorticoid receptor expression in hippocampus causing a receptor balance suitable for neuroprotection (Fig. S2). Persistent elevated corticosterone induces increased level of pycknosis in hippocampus while modulation the corticosterone level in hippocampus by corticosterone synthesis inhibitor metyrapone and withanoide A decreases the hypoxia induced elevated numbers of pycknotic cells (Fig. S3). Increased co-labelling of neuronal marker Neu N with apoptotic marker caspase 3 further indicate that the nature of cells undergoing apoptosis are neurons of hippocampal region (Fig. S4). Data expressed as percentage change taking normoxic value as 100% and represents Mean ± SEM. ‘a’ denotes p≤0.05 vs. when compared to normoxic group and ‘b’ denotes p≤0.05 vs. when compared to 7 days hypoxic group treated with vehicle only. This finding shows that elevated corticosterone or its downstream signaling play pivotal role in neuronal survivability and memory functions in hypobaric hypoxic condition. Thus modulation of corticosterone could provide therapeutic strategy to reverse hypoxia induced physiological and pathological disorders.

## Supporting Information

Figure S1 **Withanolide A modulates corticosterone level in hippocampus during hypoxic exposure.** Prolonged exposure to hypobaric hypoxia elevates hippocampal corticosterone level. Administration of withanolide A decreases the level of hippocampal corticosterone just above the normoxic level optimum for its protective effect. Data expressed as percentage change taking normoxic value as 100% and represents Mean ± SEM. ‘a’ denotes p≤0.05 vs. when compared to normoxic group and ‘b’ denotes p≤0.05 vs. when compared to 7 days hypoxic group treated with vehicle only.
(TIF)

Figure S2 **Withanolide A modulates glucocorticoid and mineralocorticoid receptor expression in hippocampus during hypoxia.** Withanolide A administration during hypoxic exposure decreases glucocorticoid receptor and increases mineralocorticoid receptor expression in hippocampus causing a receptor balance suitable for neuroprotection. Data expressed as percentage change taking normoxic value as 100% and represents Mean ± SEM. ‘a’ denotes p≤0.05 vs. when compared to normoxic group and ‘b’ denotes p≤0.05 vs. when compared to 7 days hypoxic group treated with vehicle only.
(TIF)

Figure S3 **Optimal maintainance of corticosterone level using metyrapone and withanolide A during hypoxia provide neuroprotection in hippocampus.** Withanolide A modulate the corticosterone level in hippocampus and decreases the hypoxia induced elevated pycknotic cells comparable to metyrapone. Data expressed as percentage change taking normoxic value as 100% and represents Mean ± SEM. ‘a’ denotes p≤0.05 vs. when compared to normoxic group and ‘b’ denotes p≤0.05 vs. when compared to 7 days hypoxic group treated with vehicle only.
(TIF)

Figure S4 **Representative slides showing the double labelled neuronal cells with apoptotic marker caspase 3 and neuronal marker Neu N in the CA3 region of the hippocampus.** Double labelled cells indicates the apoptotic neuronal cells.
(TIF)

## References

[1] Bahrke M, Hale BS (1993) Effect of altitude on mood, behavior and cognitive functioning. Sports Med 16: 97–125. 8378672 10.2165/00007256-199316020-00003

[2] Baitharu I, Jain V, Deep SN, Sahu JK, Naik PK, et al (2013) Exposure to hypobaric hypoxia and reoxygenation induces transient anxiety like behaviour in rat. J Behav Brain Sci 3: 591–602.

[3] Won SJ, Kim DY, Gwag BJ (2002) Cellular and molecular pathways of ischemic neuronal death. J Biochem Mol Biol 35: 67–86. 16248972 10.5483/bmbrep.2002.35.1.067

[4] Barker JE, Heales SJR, Cassidy A, Bolanos JP, Land JM, et al (1996) Depletion of brain glutathione results in a decrease in glutathione reductase activity, an enzyme susceptible to oxidative damage. Brain Res 716: 118–122. 8738227 10.1016/0006-8993(96)00003-0

[5] Maiti P, Singh SB, Sharma AK, Muthuraju S, Banerjee PK, et al (2006) Hypobaric hypoxia induces oxidative stress in rat brain. Neurochem International 49: 709–716. 10.1016/j.neuint.2006.06.002 16911847

[6] Moller P, Loft S, Lundby C, Olsen NV (2001) Acute hypoxia and hypoxic exercise induced DNA strand breaks and oxidative DNA damage in humans. FASEB J 15: 1181–1186. 11344086 10.1096/fj.00-0703com

[7] Chandel NS, Maltepe E, Goldwasser E, Mathieu CE, Simon MC, et al (1998) Mitochondrial reactive oxygen species trigger hypoxia-induced transcription. Proc Natl Acad Sci U S A. 95: 11715–11720. 9751731 10.1073/pnas.95.20.11715 PMC21706

[8] Hota SK, Barhwal K, Singh SB, Ilavazhagan G (2007) Differential temporal response of hippocampus, cortex and cerebellum to hypobaric hypoxia: a biochemical approach. Neurochem International 51: 384–390. 10.1016/j.neuint.2007.04.003 17531352

[9] Jayalakshmi K, Singh SB, Kalpana B, Sairam M, Muthuraju S, et al (2007) N-acetyl cysteine supplementation prevents impairment of spatial working memory functions in rats following exposure to hypobaric hypoxia. Physiol Behav 92: 643–650. 17602713 10.1016/j.physbeh.2007.05.051

[10] Prasad J, Baitharu I, Sharma AK, Dutta R, Prasad D, et al (2013) Quercetin reverses hypobaric hypoxia-induced hippocampal neurodegeneration and improves memory function in the rat. High Altitude Med Biol 14: 383–394. 10.1089/ham.2013.1014 24377346

[11] Dringen R (2000) Glutathione metabolism and oxidative stress in neurodegeneration. European J Biochem 267: 4903. 10931171 10.1046/j.1432-1327.2000.01651.x

[12] Richman PG, Meister A (1975) Regulation of gamma-glutamylcysteine synthetase by nonallosteric feedback inhibition by glutathione. J Biol Chem 250: 1422–1426. 1112810

[13] Nguyen T, Nioi P, Pickett CB (2009) The Nrf2-antioxidant response element signaling pathway and its activation by oxidative stress. J Biol Chem 284: 13291–13295. 19182219 10.1074/jbc.R900010200 PMC2679427

[14] Kensler TW, Wakabayashi N, Biswal S (2007) Cell survival responses to environmental stresses via the Keap1–Nrf2–ARE pathway. Ann Rev Pharmacol and Toxicol 47: 89–116. 16968214 10.1146/annurev.pharmtox.46.120604.141046

[15] Hussain S, Slikker W Jr, Ali SF (1996) Role of metallothionein and other antioxidants in scavenging superoxide radicals and their possible role in neuroprotection. Neurochem International 29: 145–152. 10.1016/0197-0186(95)00114-x 8837043

[16] Shih AY, Imbeault S, Barakauskas V, Erb H, Jiang L, et al (2005) Induction of the Nrf2-driven antioxidant response confers neuroprotection during mitochondrial stress in vivo. J Biol Chem 280: 22925–22936. 15840590 10.1074/jbc.M414635200

[17] Baitharu I, Jain V, Deep SN, Hota KB, Hota SK, et al (2013) *Withania somnifera* root extract ameliorates hypobaric hypoxia induced memory impairment in rats. J Ethnopharmacol 145: 431–441. 23211660 10.1016/j.jep.2012.10.063

[18] Cornford EM, Braun LD, Crane PD, Oldendorf WH (1978) Blood-brain barrier restriction of peptides and the low uptake of enkephalins. Endocrinol 103: 1297–1303. 10.1210/endo-103-4-1297 744146

[19] Kannan R, Yi JR, Tang D, Li Y, Zlokovic BV, et al (1996) Evidence for the existence of a sodium-dependent glutathione (GSH) transporter. Expression of bovine brain capillary mRNA and size fractions in Xenopus laevis oocytes and dissociation from gamma-glutamyltranspeptidase and facilitative GSH transporters. J Biol Chem 271: 9754–9758. 8621654 10.1074/jbc.271.16.9754

[20] Dhuley JN (1998) Effect of ashwagandha on lipid peroxidation in stress-induced animals. J Ethnopharmacol 60: 173–178. 9582008 10.1016/s0378-8741(97)00151-7

[21] Naidu PS, Singh A, Kulkarni SK (2006) Effect of *Withania somnifera* root extract on reserpine-induced orofacial dyskinesia and cognitive dysfunction. Phytotherapy Res 220: 140–146. 10.1002/ptr.1823 16444668

[22] Schliebs R, Liebmann A, Bhattacharya SK, Kumar A, Ghosal S, et al (1997) Systemic administration of defined extracts from *Withania somnifera* (Indian Ginseng) and Shilajit differentially affects cholinergic but not glutamatergic and GABAergic markers in rat brain. Neurochem International 30: 181–190. 10.1016/s0197-0186(96)00025-3 9017665

[23] Tohda C, Kuboyama T, Komatsu K (2000) Dendrite extension by methanol extract of Ashwagandha (roots of *Withania somnifera*) in SK-N-SH cells. Neuroreport 11: 1981–1985. 10884056 10.1097/00001756-200006260-00035

[24] Kuboyama T, Tohda C, Zhao J, Nakamura N, Hattori M, et al (2002) Axon- or dendrite-predominant outgrowth induced by constituents from Ashwagandha. Neuroreport 13: 1715–20. 12395110 10.1097/00001756-200210070-00005

[25] Hota SK, Barhwal K, Baitharu I, Prasad D, Singh SB, et al (2009) Bacopa monniera leaf extract ameliorates hypobaric hypoxia induced spatial memory impairment. Neurobiol Dis 34: 23–39. 19154788 10.1016/j.nbd.2008.12.006

[26] Barhwal K, Hota SK, Baitharu I, Prasad D, Singh SB, et al (2009) Isradipine antagonizes hypobaric hypoxia induced CA1 damage and memory impairment: complementary roles of L-type calcium channel and NMDA receptors. Neurobiol Dis 34: 230–244. 19385055 10.1016/j.nbd.2009.01.008

[27] Ando D, Yamakita M, Yamagata Z, Koyama K (2009) Effects of glutathione depletion on hypoxia-induced erythropoietin production in rats. J Physiol Anthropol 28: 211–215. 19823002 10.2114/jpa2.28.211

[28] Baitharu I, Deep SN, Jain V, Barhwal K, Malhotra AS, et al (2011) Corticosterone synthesis inhibitor metyrapone ameliorates chronic hypobaric hypoxia induced memory impairment in rat. Behav Brain Res 228: 53–65. 22137888 10.1016/j.bbr.2011.11.030

[29] Smith-Swintosky VL, Pettigrew LC, Sapolsky RM, Phares C, Craddock SD, et al (1996) Metyrapone, an inhibitor of glucocorticoid production, reduces brain injury induced by focal and global ischemia and seizures. J Cerebral Blood Flow and Metabol 16: 585–598. 10.1097/00004647-199607000-00008 8964797

[30] Bradford MM (1976) A rapid and sensitive method for the quantitation of microgram quantities of protein utilizing the principle of protein-dye binding. Anal Biochem 72: 248–254. 942051 10.1016/0003-2697(76)90527-3

[31] LeBel CP, Ali SF, McKee M, Bondy SC (1990) Organometal-induced increases in oxygen reactive species: the potential of 2,7-dichlorofluorescin diacetate as an index of neurotoxic damage. Toxicol Applied Pharmacol 104: 17–24. 10.1016/0041-008x(90)90278-3 2163122

[32] Myhre O, Andersen JM, Aarnes H, Fonnum F (2003) Evaluation of the probes 2′, 7′-dichlorofluorescin diacetate, luminol, and lucigenin as indicators of reactive species formation. Biochem Pharmacol 65: 1575–1582. 12754093 10.1016/s0006-2952(03)00083-2

[33] Das NP, Ratty AK (1987) Studies on the effects of the narcotic alkaloids, cocaine, morphine, and codeine on nonenzymatic lipid peroxidation in rat brain mitochondria. Biochem Med Metab Biol 37: 258–264. 3593597 10.1016/0885-4505(87)90035-1

[34] Colado MI, OShea E, Granaburn litvakdos R, Murray TK, Green AR (1997) In vivo evidence for free radical involvement in the degeneration of rat brain 5-HT following administration of MDMA and p-chloroamphetamine but not the degeneration following fenfluramine. Br J Pharmacol 121: 889–900. 9222545 10.1038/sj.bjp.0701213 PMC1564770

[35] Hissin PJ, Hilf R (1976) A fluorimetric method for determination of oxidized and GSH in tissue. Anal Biochem 74: 214–226. 962076 10.1016/0003-2697(76)90326-2

[36] Pinto RE, Bartley W (1969) The effect of age and sex on glutathione reductase and glutathione peroxidase activities and on aerobic glutathione oxidation in rat liver homogenates. Biochem J 112: 109–115. 4388243 10.1042/bj1120109 PMC1187646

[37] Mishra A, Roy KP (2006) 2-OHE2-induced oocyte maturation involves steroidogenesis in cat fish. J Endocrinol 189: 341–353.16648301

[38] Seelig GF, Meister A (1984) γ-Glutamylcysteine synthetase. Interactions of an essential sulfhydryl group. J Biol Chem 259: 3534–3538. 6142890

[39] Morris RGM (1984) Development of a water maze procedure for studying spatial learning the rat. J Neurosci Method 11: 47–60. 10.1016/0165-0270(84)90007-4 6471907

[40] Baitharu I, Deep SN, Jain V, Prasad D, Ilavazhagan G (2013) Inhibition of glucocorticoid receptors ameliorates hypobaric hypoxia induced memory impairment in rat. Behav Brain Res 240: 76–86. 23159706 10.1016/j.bbr.2012.11.005

[41] Winterbourn CC, Metodiewa D (1994) The reaction of superoxide with GSH. Arch Biochem Biophyics 314: 284–290. 10.1006/abbi.1994.1444 7979367

[42] Clancy RM, Levartovsky D, Leszczynska-Piziak J, Yegudin J, Abramson SB (1994) Nitric oxide reacts with intracellular glutathione and activates the hexose monophosphate shunt in human neutrophils: evidence for S-nitrosoglutathione as a bioactive intermediary. Proc Natl Acad Sci U S A 91: 3680–3684. 8170969 10.1073/pnas.91.9.3680 PMC43645

[43] Bains JS, Shaw CA (1997) Neurodegenerative disorders in humans: the role of glutathione in oxidative stress-mediated neuronal death. Brain Res Rev 25: 335–358. 9495562 10.1016/s0165-0173(97)00045-3

[44] Giustarini D, Rossi R, Milzan A, Colombo R, Dalle-Donne I (2004) S-glutathionylation: from redox regulation of protein functions to human diseases. J Cell Mol Med 8: 201–212. 15256068 10.1111/j.1582-4934.2004.tb00275.x PMC6740303

[45] Janaky R, Ogita K, Pasqualotto BA, Bains JS, Oja SS, et al (1999) Glutathione and signal transduction in the mammalian CNS. J Neurochem 73: 889–902. 10461878 10.1046/j.1471-4159.1999.0730889.x

[46] Cooper AJ, Kristal BS (1997) Multiple roles of glutathione in the central nervous system. Biol Chem 378: 793–802. 9377474

[47] Bhattacharya A, Ghosal S, Bhattacharya SK (2001) Anti-oxidant effect of Withania somnifera glycowithanolides in chronic footshock stress-induced perturbations of oxidative free radical scavenging enzymes and lipid peroxidation in rat frontal cortex and striatum. J Ethnopharmacol 74: 1–6. 11137343 10.1016/s0378-8741(00)00309-3

[48] Anwer T, Sharma M, Pillai KK, Khan G (2012) Protective effect of *Withania somnifera* against oxidative stress and pancreatic beta-cell damage in type 2 diabetic rats. Acta Polon Pharmacol 69: 1095–1101. 23285670

[49] Parihar MS, Hemnani T (2003) Phenolic antioxidants attenuate hippocampal neuronal cell damage against kainic acid induced excitotoxicity. J Biosci 28: 121–128. 12682435 10.1007/BF02970142

[50] Gupta A, Gupta A, Datta M, Shukla GS (2000) Cerebral antioxidant status and free radical generation following glutathione depletion and subsequent recovery. Mol Cel Biochem 209: 55–61. 10.1023/a:1007000430394 10942201

[51] Kim JY, Yim JH, Cho JH, Kim JH, Ko JH, et al (2006) Adrenomedullin regulates cellular glutathione content via modulation of gamma-glutamate-cysteine ligase catalytic subunit expression. Endocrinol 147: 1357–1364. 10.1210/en.2005-0895 16322067

[52] Yang YC, Lii CK, Lin AH, Yeh YW, Yao HT, et al (2011) Induction of glutathione synthesis and heme oxygenase 1 by the flavonoids butein and phloretin is mediated through the ERK/Nrf2 pathway and protects against oxidative stress. Free Rad Biol Med 51: 2073–2081. 21964506 10.1016/j.freeradbiomed.2011.09.007

[53] Blum J, Fridovich I (1985) Inactivation of glutathione peroxidase by superoxide radical. Arch Biochem Biophysics 240: 500–508. 10.1016/0003-9861(85)90056-6 2992378

[54] Sharma R, Yang Y, Sharma A, Awasthi S, Awasthi YC (2004) Antioxidant role of glutathione S-transferases: protection against oxidant toxicity and regulation of stress-mediated apoptosis. Antioxidant Red Signal 6: 289–300. 10.1089/152308604322899350 15025930

[55] Beckman JS, Beckman TW, Chen J, Marshall PA, Freeman BA (1990) Apparent hydroxyl radical production by peroxynitrite: implications for endothelial injury from nitric oxide and superoxide. Proc Natl Acad Sci USA 87: 1620–1624. 2154753 10.1073/pnas.87.4.1620 PMC53527

[56] Miki N, Kawabe Y, Kuriyama K (1977) Activation of cerebral guanylate cyclase by nitric oxide. Biochem Biophysical Res Comm 75: 851–856. 10.1016/0006-291x(77)91460-7 16602

[57] Singh RJ, Hogg N, Joseph J, Kalyanaraman B (1996) Mechanism of nitric oxide release from S-nitrosothiols. J Biol Chem 271: 18596–18603. 8702510 10.1074/jbc.271.31.18596

[58] Rauhala P, Lin AM, Chiueh CC (1998) Neuroprotection by S-nitrosoglutathione of brain dopamine neurons from oxidative stress. FASEB J 12: 165–173. 9472981 10.1096/fasebj.12.2.165

[59] Garcia-Ruiz JC, Fernández-Checa C (2007) Redox regulation of hepatocyte apoptosis. J Gastroenterol Hepatol 22: S38–S42. 17567462 10.1111/j.1440-1746.2006.04644.x

[60] Maiti P, Singh SB, Mallick B, Muthuraju S, Ilavazhagan G (2008) High altitude memory impairment is due to neuronal apoptosis in hippocampus, cortex and striatum. J Chem Neuroanatom 36: 227–238.la. 10.1016/j.jchemneu.2008.07.003 18692566

[61] Mizui T, Kinouchi H, Chan PH (1992) Depletion of brain glutathione by buthionine sulfoximine enhances cerebral ischemic injury in rats. Am J Physiol 262: H313–317. 1539690 10.1152/ajpheart.1992.262.2.H313

[62] Wuellner U, Seyfried J, Groscurth P, Beinroth S, Winter S, et al (1999) Glutathione depletion and neuronal cell death: the role of reactive oxygen intermediates and mitochondrial function. Brain Res 826: 53–62. 10216196 10.1016/s0006-8993(99)01228-7

[63] Pileblad E, Magnusson T, Fornstedt B (1989) Reduction of brain glutathione by L-buthionine sulfoximine potentiates the dopamine-depleting action of 6-hydroxydopamine in rat striatum. J Neurochem 52: 978–980. 2493072 10.1111/j.1471-4159.1989.tb02550.x

[64] Ferrari R, Ceconi C, Curello S, Cargnoni A, Alfieri O, et al (1991) Oxygen free radicals and myocardial damage: protective role of thiol-containing agents. Am J Med 91: 95S–105S. Review. 10.1016/0002-9343(91)90291-5 1928219

[65] Choy KH, Dean O, Berk M, Bush AI, van den Buuse M (2010) Effects of N-acetyl-cysteine treatment on glutathione depletion and a short-term spatial memory deficit in 2-cyclohexene-1-onetreated rats. European J Pharmacol 649: 224–228. 20868666 10.1016/j.ejphar.2010.09.035

[66] Bhatnagar M, Sharma D, Salvi M (2009) Neuroprotective effects of *Withania somnifera* dunal.: a possible mechanism. Neurochem Res 34: 1975–1983. 19444606 10.1007/s11064-009-9987-7

[67] Kratschmar DV, Calabrese D, Walsh J, Lister A, Birk J, et al (2012) Suppression of the Nrf2-Dependent Antioxidant Response by Glucocorticoids and 11b- HSD1-Mediated Glucocorticoid Activation in Hepatic Cells. PLoS ONE 7, e36774. 22606287 10.1371/journal.pone.0036774 PMC3350474

[68] Ki SH, Cho IJ, Choi DW, Kim SG (2005) Glucocorticoid receptor (GR)- associated SMRT binding to C/EBPbeta TAD and Nrf2 Neh4/5: role of SMRT recruited to GR in GSTA2 gene repression. Mol Cel Biol 25: 4150–4165. 10.1128/MCB.25.10.4150-4165.2005 PMC1087722 15870285

