# Supplementary figures and images for: Withanolide A Prevents Neurodegeneration by Modulating Hippocampal Glutathione Biosynthesis during Hypoxia
Source: PLoS One. 2014 Oct 13;9(10):e105311. doi: 10.1371/journal.pone.0105311 (PMC4195593; doi:10.1371/journal.pone.0105311)

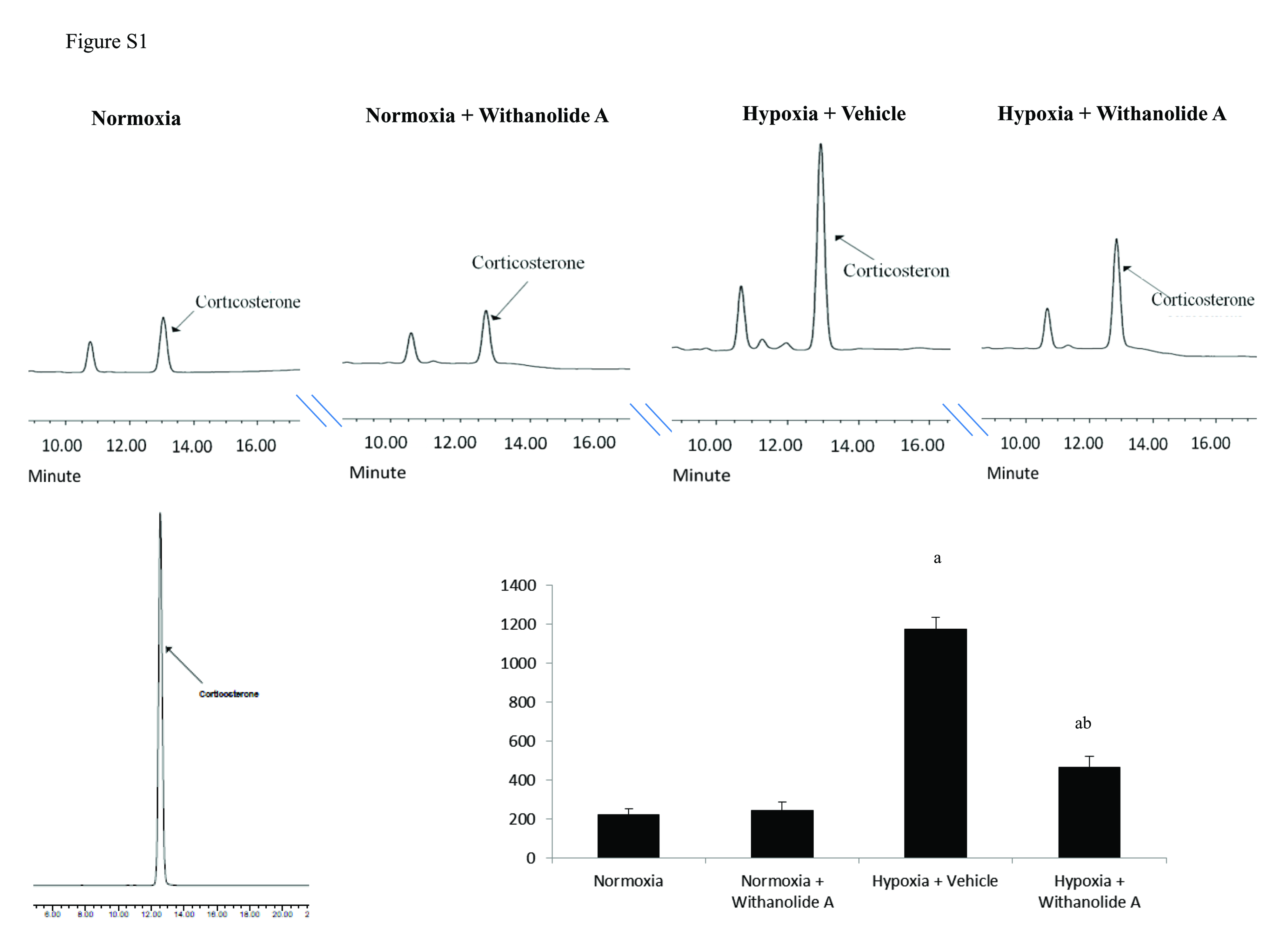

Supplement: Figure S1 — Withanolide A modulates corticosterone level in hippocampus during hypoxic exposure. Prolonged exposure to hypobaric hypoxia elevates hippocampal corticosterone level. Administration of withanolide A decreases the level of hippocampal corticosterone just above the normoxic level optimum for its protective effect. Data expressed as percentage change taking normoxic value as 100% and represents Mean ± SEM. ‘a’ denotes p≤0.05 vs. when compared to normoxic group and ‘b’ denotes p≤0.05 vs. when compared to 7 days hypoxic group treated with vehicle only. (TIF) [file pone.0105311.s001.tif]

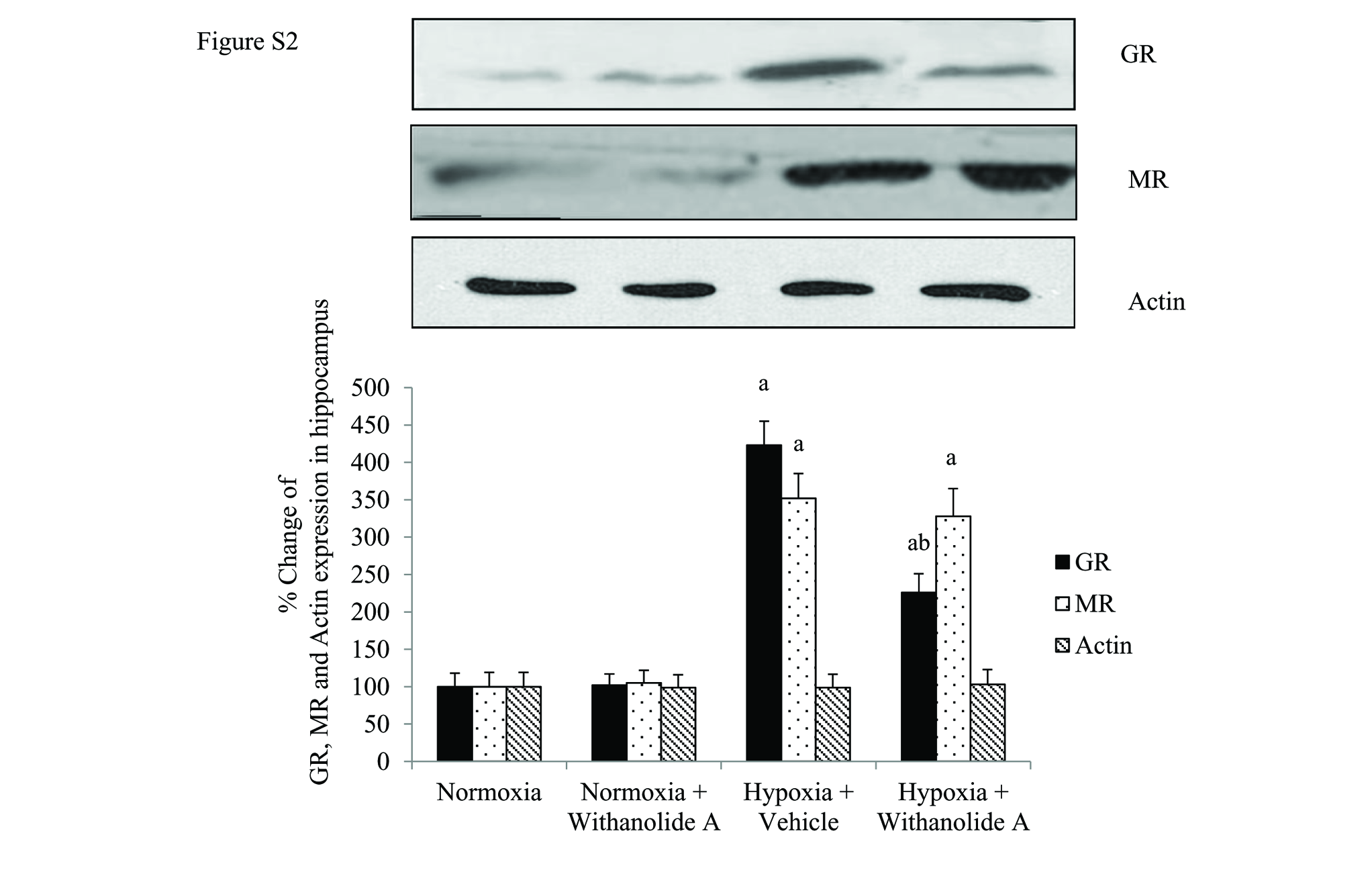

Supplement: Figure S2 — Withanolide A modulates glucocorticoid and mineralocorticoid receptor expression in hippocampus during hypoxia. Withanolide A administration during hypoxic exposure decreases glucocorticoid receptor and increases mineralocorticoid receptor expression in hippocampus causing a receptor balance suitable for neuroprotection. Data expressed as percentage change taking normoxic value as 100% and represents Mean ± SEM. ‘a’ denotes p≤0.05 vs. when compared to normoxic group and ‘b’ denotes p≤0.05 vs. when compared to 7 days hypoxic group treated with vehicle only. (TIF) [file pone.0105311.s002.tif]

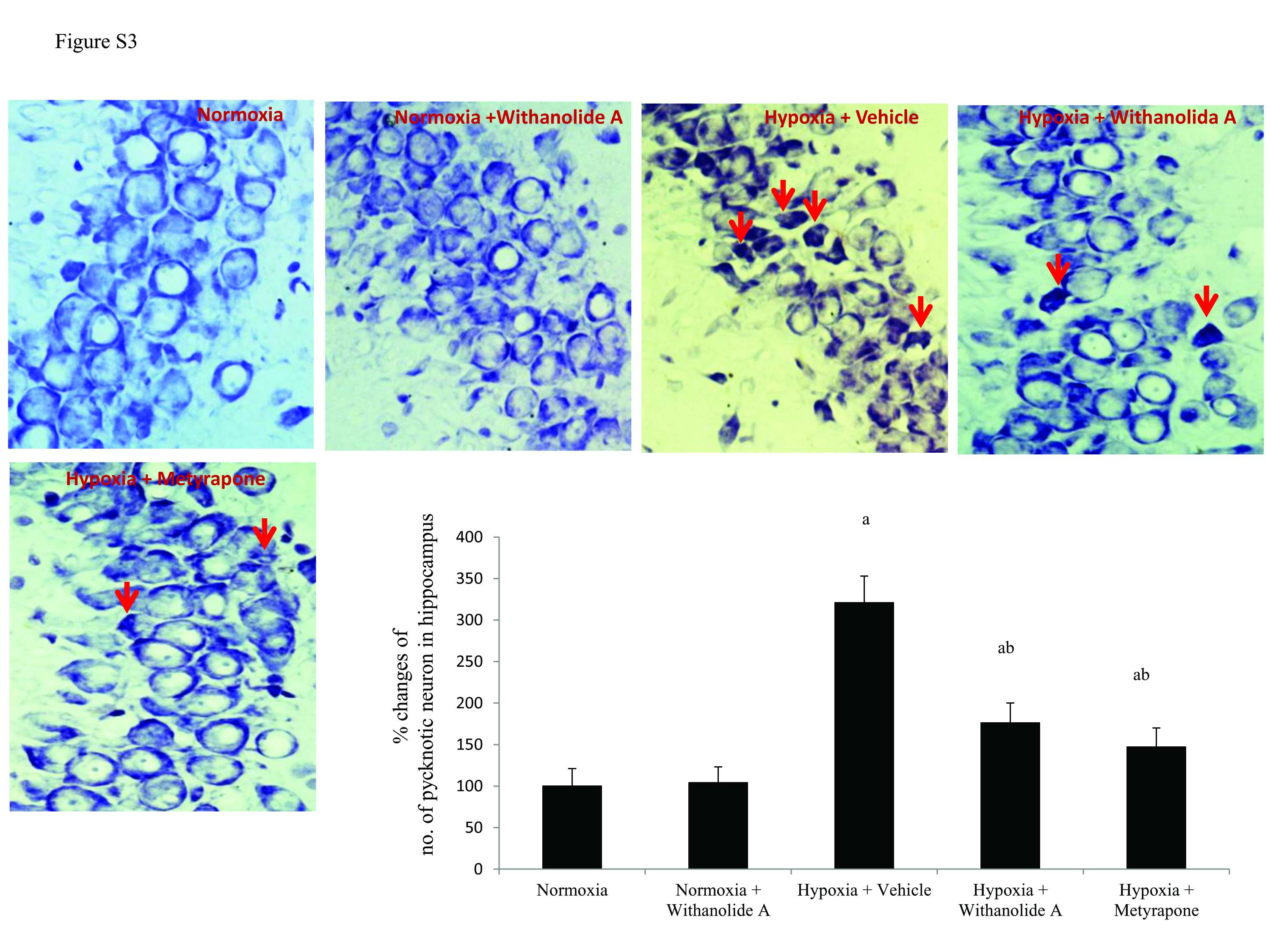

Supplement: Figure S3 — Optimal maintainance of corticosterone level using metyrapone and withanolide A during hypoxia provide neuroprotection in hippocampus. Withanolide A modulate the corticosterone level in hippocampus and decreases the hypoxia induced elevated pycknotic cells comparable to metyrapone. Data expressed as percentage change taking normoxic value as 100% and represents Mean ± SEM. ‘a’ denotes p≤0.05 vs. when compared to normoxic group and ‘b’ denotes p≤0.05 vs. when compared to 7 days hypoxic group treated with vehicle only. (TIF) [file pone.0105311.s003.tif]

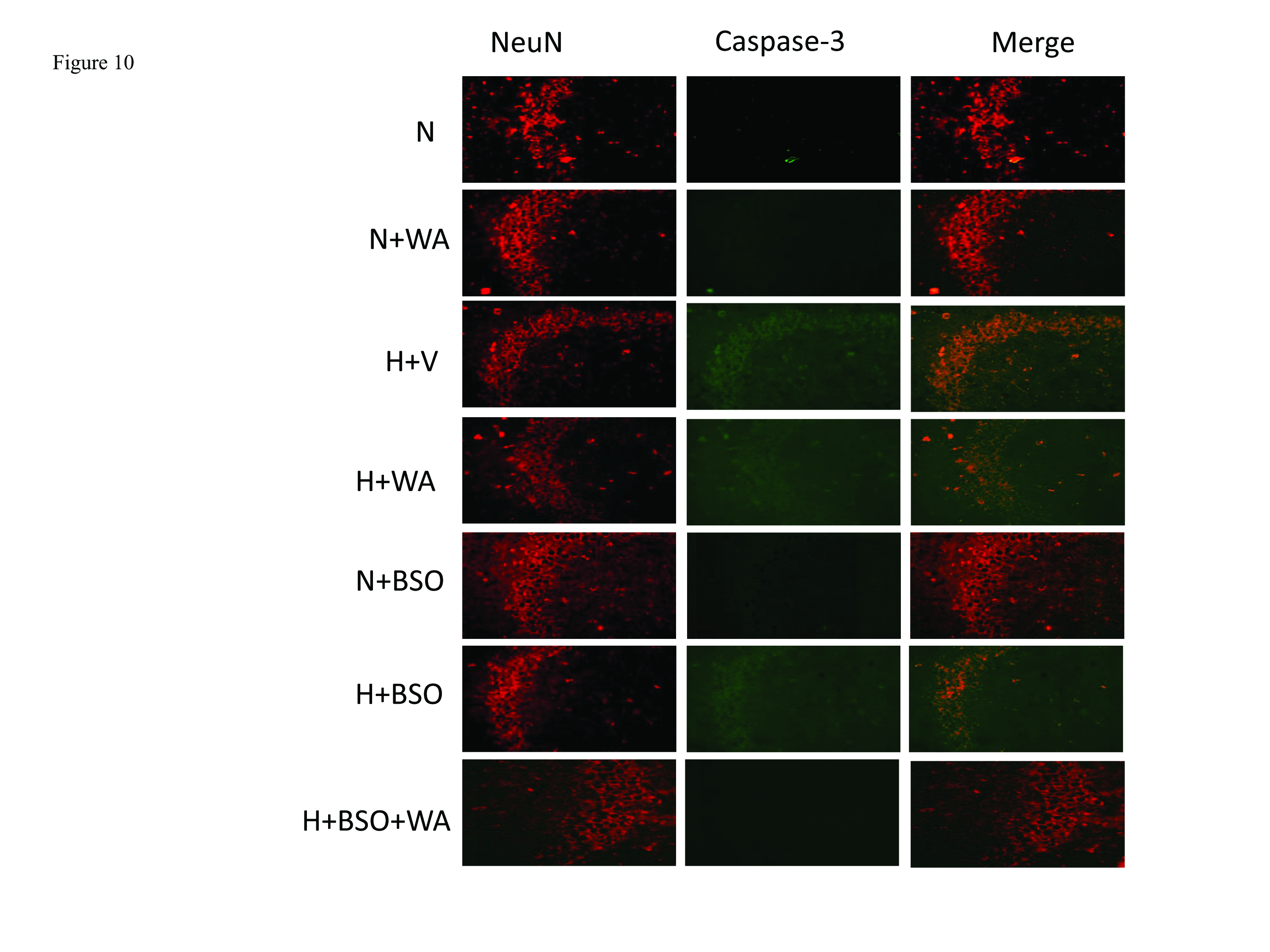

Supplement: Figure S4 — Representative slides showing the double labelled neuronal cells with apoptotic marker caspase 3 and neuronal marker Neu N in the CA3 region of the hippocampus. Double labelled cells indicates the apoptotic neuronal cells. (TIF) [file pone.0105311.s004.tif]
